# A large-scale Boolean model of the rheumatoid arthritis fibroblast-like synoviocytes predicts drug synergies in the arthritic joint

**DOI:** 10.1038/s41540-023-00294-5

**Published:** 2023-07-15

**Authors:** Vidisha Singh, Aurelien Naldi, Sylvain Soliman, Anna Niarakis

**Affiliations:** 1 Université Paris-Saclay, Laboratoire Européen de Recherche pour la Polyarthrite rhumatoïde—Genhotel, Univ Evry, Evry, France; 2 Lifeware Group, Inria, Saclay-île de France, 91120 Palaiseau, France

**Keywords:** Virtual drug screening, Dynamic networks

## Abstract

Rheumatoid arthritis (RA) is a complex autoimmune disease with an unknown aetiology. However, rheumatoid arthritis fibroblast-like synoviocytes (RA-FLS) play a significant role in initiating and perpetuating destructive joint inflammation by expressing immuno-modulating cytokines, adhesion molecules, and matrix remodelling enzymes. In addition, RA-FLS are primary drivers of inflammation, displaying high proliferative rates and an apoptosis-resistant phenotype. Thus, RA-FLS-directed therapies could become a complementary approach to immune-directed therapies by predicting the optimal conditions that would favour RA-FLS apoptosis, limit inflammation, slow the proliferation rate and minimise bone erosion and cartilage destruction. In this paper, we present a large-scale Boolean model for RA-FLS that consists of five submodels focusing on apoptosis, cell proliferation, matrix degradation, bone erosion and inflammation. The five-phenotype-specific submodels can be simulated independently or as a global model. In silico simulations and perturbations reproduced the expected biological behaviour of the system under defined initial conditions and input values. The model was then used to mimic the effect of mono or combined therapeutic treatments and predict novel targets and drug candidates through drug repurposing analysis.

## Introduction

Rheumatoid arthritis (RA) is a multifactorial disease that affects the articular joints of the human body and involves combinations of genetic factors and environmental triggers^1–3^. Cytokines such as tumour necrosis factor-alpha (TNF-α) and interleukin 6 (IL-6) are central players in RA, causing inflammation and tissue damage, whereas their inhibitors are considered among the main treatments for RA^2,4,5^. However, despite a growing number of such drugs, 40% of patients fail to respond to therapy adequately^6,7^. Recent studies have identified RA fibroblast-like synoviocytes (RA-FLS) as responsible for up to a quarter of the disease’s heritability^8^, attributing to these cells a causal role in disease pathogenesis. The primary roles of RA-FLS in RA are discussed below^9,10^. RA-FLS plays a central role in the pathogenesis of RA by activating the innate immune response. The immune response is maintained via the secretion of soluble molecules, such as proinflammatory cytokines IL-6, IL-1 and TNF, in response to environmental stimuli and interactions with other cells. Secretions of these molecules act as a positive feedback loop and eventually trigger the activation of RA-FLS into expressing the responses repeatedly^9^. Joint inflammation is the primary characteristic of RA. During joint inflammation in RA, FLS proliferate to form the pannus, which invades and destroys the cartilage. Major pathways involved in the perpetuation of inflammation are TNF, IL-6, IL-1 and IL-17^4,11^.

The final stages of the disease involve the degradation of bone and cartilage due to chronic inflammation within the joint area. During chronic and sustained inflammation, RA-FLS secrete two groups of soluble molecules: (a) receptor activator of nuclear factor kappa-Β ligand (RANKL), a molecule that promotes osteoblasts differentiation to osteoclasts, cells that are responsible for bone degradation and bone resorption^12,13^ and (b) matrix metalloproteinases (MMPs), a group of matrix proteases responsible for the degradation and breakdown of numerous extracellular matrix components such as collagen, leading to the degradation of cartilage^14,15^. The synergistic activity of these factors (RANKL and MMPs) leads to the gradual degradation of bone and cartilage in the joint area, leading to stiffness, pain and eventually disability of movement. Lastly, RA-FLS aberrant proliferation contributes to pannus formation and joint destruction. RA-FLS are the critical cell types in the growth of pathological synovial tissue in RA, and inhibition of their proliferation is a potential antirheumatic therapy^16^. RA-FLS are shown to be apoptosis-resistant^17^. The intrinsic and extrinsic pathways regulate apoptosis. The intrinsic pathway is activated inside the cell following intracellular stress or injury, including mitochondrial proteins like BH3 interacting domain death agonist (BID), Bcl-2-associated X protein (BAX), B-cell lymphoma 2 protein (BCL2), and phorbol-12-myristate-13-acetate-induced protein 1 (PMAIP1), among others. The tumour protein P53 (TP53) transcription factor is a critical activator of the intrinsic pathway of apoptosis via activating mitochondrial proteins. TNF and FAS ligand (FASLG) mainly activate the extrinsic pathway. Ultimately, both pathways activate caspases, which initiate a proteolytic cascade leading to cellular death^18^.

The biological information regarding pathways implicated in the RA-FLS pathology has been assembled into a large-scale mechanistic network, the RA map^19^. While the RA map contains generic information about pathways implicated in RA that come from various sources, the RA-FLS is the dominant cell type. The map is a process description diagram^20^ and is part of the Disease Maps initiative^21,22^. This type of construct contains valuable disease-specific information encoded in human and machine-readable formats. Besides serving as templates for omic data visualisation, disease maps can also work as scaffolds for building mathematical models and sources of causal interactions^23^.

Dynamical modelling has been widely used to study and decipher complex biological processes that are otherwise hard to comprehend. Previous attempts to build computational models for RA have contributed a few kinetic models to study the role of proinflammatory and anti-inflammatory cytokines^24^ and bone erosion^25^, and also the behaviour of various cells, including RA-FLS in cartilage destruction in RA joints^26^. A hybrid mathematical modelling framework that describes pannus production in a tiny proximal interphalangeal (PIP) joint was also proposed^27^. Nevertheless, the need for kinetic parameters limits its use for large-scale molecular interaction networks. Lastly, a large-scale hybrid model covering signalling, gene regulation, and metabolism in RA-FLS was published^28^. The model focuses on the metabolic reprogramming of fibroblasts under hypoxic conditions in the arthritic joint and suggests a reverse Warburg effect as the origin of the observed metabolic switch.

Discrete logic-based qualitative modelling has been increasingly used to model large-scale networks for which kinetic data is scarce^29,30^. For example, in a Boolean model, each node can take only two values, 0 (FALSE) and 1 (TRUE). The next value of each variable is determined by a logical function (using the classical AND, OR, NOT operators) of the current values of its regulators (upstream nodes). The evolution of each node also depends on the updating scheme chosen. The synchronous scheme updates all variables in the model simultaneously; in the asynchronous scheme, the variables are updated individually in a non-synchronous manner^31,32^.

**Fig. 1:**
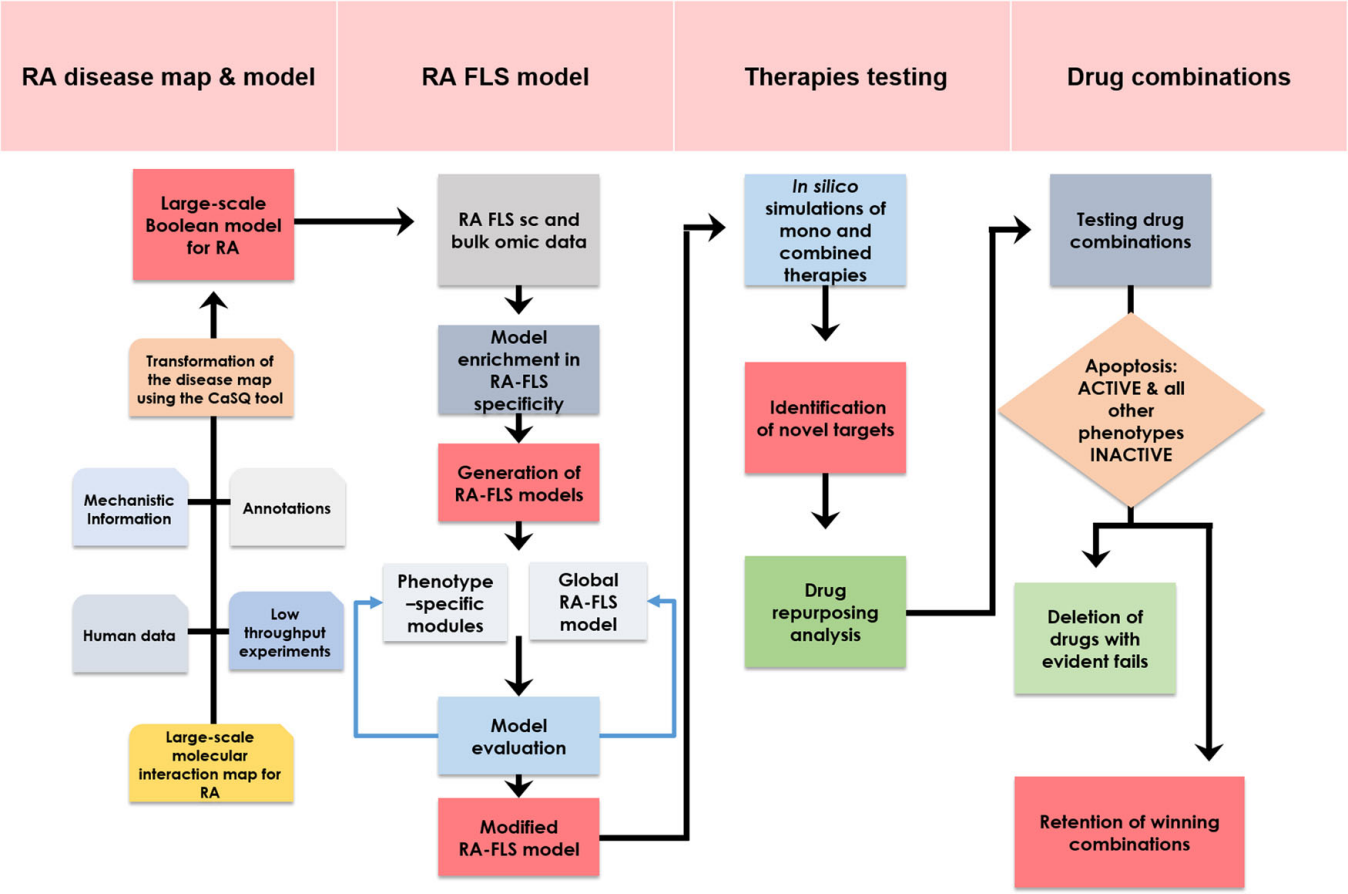
Construction and simulation of a large-scale, modular Boolean model of RA-FLS for evaluating novel drug combinations. The RA map was converted into an executable Boolean model using the map-to-model framework described in ref. ^33^. Using single-cell omic datasets and literature studies, the RA generic model was subsequently enriched in RA-FLS-specific data. The RA-FLS model focuses on five phenotypes (apoptosis, cell proliferation, inflammation, matrix degradation, and bone erosion) characteristic of RA’s fibroblasts. Individual phenotype-specific submodels and a five-phenotype global model were created. Biological scenarios extracted from the literature were used to evaluate and validate the models’ behaviour leading to some modifications of the original models. The modified RA-FLS model was then used to test mono and combined RA therapies. Drug repurposing analysis and further drug combination simulations led to a panel of suggestions of drug combinations that are predicted to have a favourable outcome (apoptosis active, cell proliferation, inflammation, bone erosion and matrix degradation inactive).

In this work, we use the state-of-the-art RA map^19^ and the tool CaSQ^33^ to build a large-scale, modular Boolean model of RA-FLS focused on inflammation, bone erosion, cartilage destruction, cell proliferation, and apoptosis. The model consists of five-phenotype-specific submodels (to be mentioned as *modules* later in the paper) that can be simulated individually and a five-phenotype global model (to be mentioned as the *global model* later in the paper). Systematic testing of different initial conditions using the modules and the global model showed that both the modules and the global model could reproduce small-scale experimental results from the literature. To search for meaningful steady states, we reduce calculations by propagating and eliminating fixed input components, as introduced in ref. ^34^ and implemented in the CoLoMoTo notebook^35^ in ref. ^36^. Input propagation consists of assigning fixed values to some of the model’s inputs and using Boolean algebra to simplify the rules of their downstream components. We also use a probabilistic framework to calculate phenotypic probabilities starting from predefined initial conditions. The RA-FLS model was used to study the effects of mono and combined therapy for RA and suggest potential targets that could enhance the desired phenotypic outcome. Furthermore, drug repurposing analysis identified possible drug candidates that have as targets the previously identified nodes. A new round of simulations was then performed to evaluate their impact on the cellular phenotype and, subsequently, on the arthritic joint (Fig. 1).

## Results

### Enhancing the cell specificity of the initial network

RA-FLS is the most frequent cell type in the RA map, covering a total of 45%, followed by synovial tissue with 36%^19^. We used the updated fibroblast overlay list provided in refs. ^37,38^ to calculate the RA-FLS specificity of the global model. In this study, they used the RA fibroblast list provided in ref. ^19^ and updated it with the RNA-seq single-cell dataset available in the GEO database^39^ by performing Differential Expression Analysis (DEA) using BioTuring software and the Venice method. Using this list, out of 261 unique model components (proteins, genes, phenotypes), 194 are RA-FLS-specific. Other cell types primarily include synovial tissue with 29 components, peripheral blood mononuclear cells (PBMCs) with 25 components, along with the presence of other sources like blood, synovial fluid, T-helper (th1), macrophages and chondrocytes (Supplementary Table 1).

### Five-phenotype-specific modules and global RA-FLS model

**Fig. 2:**
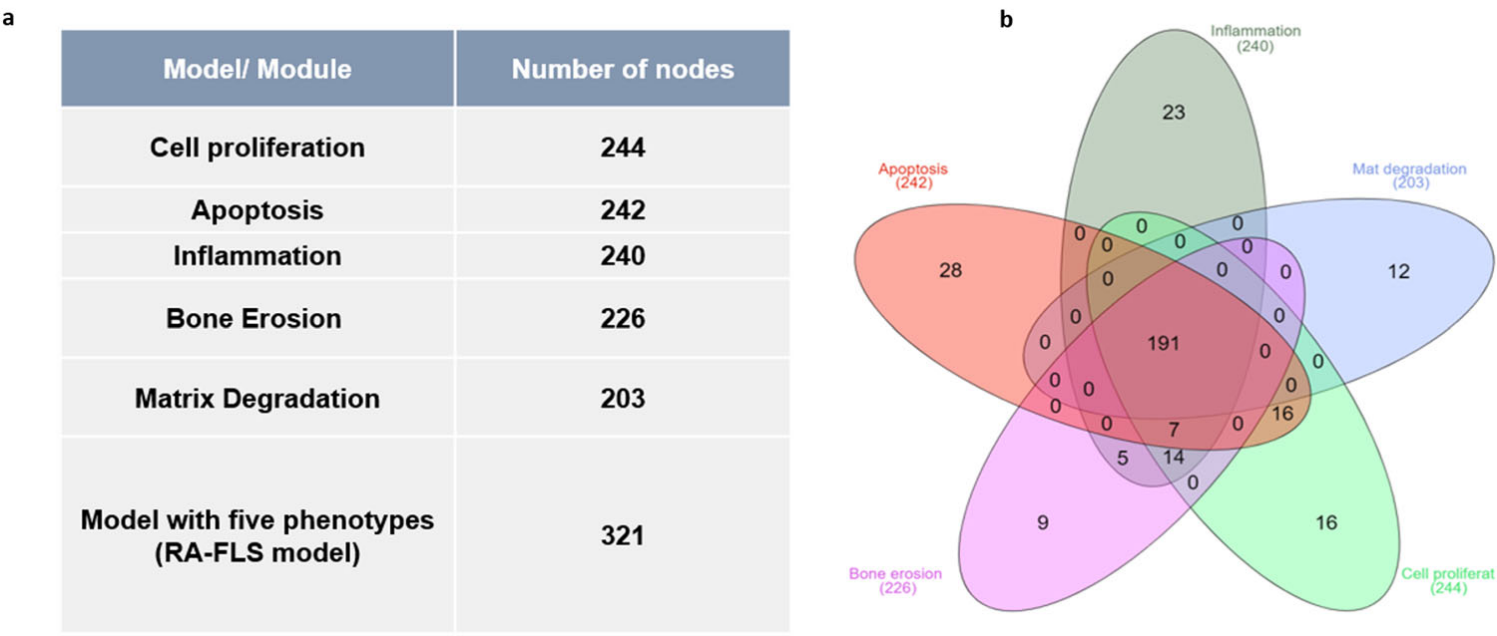
Number of nodes per module/model and shared components among modules. **a** Number of nodes of the phenotype-specific modules and the global model. **b** Venn diagram of all the five-phenotype-specific components. The core of 191 nodes is shared among all five modules, and only a few are characteristic of the corresponding phenotype-specific module.

The tool bioLQM^40^ was used to extract the phenotype-specific modules and the global model using the SBML file produced by CaSQ^33^ using RA map (Supplementary Fig. 1) XML file as input (files available on GitLab). The size of the models in terms of nodes and reactions can be seen in Fig. 2a. One straightforward observation is that the size of the five-phenotype global model is not the sum of the subparts. Indeed, as seen in the Venn diagram (Fig. 2b), there is a core of 191 nodes shared by all modules, and only a few nodes are characteristic of each phenotype-specific module.

### Evaluating the RA-FLS model under different sets of input configurations using the input propagation method

The validation of each phenotype-specific module and the global model was performed by comparing the outcome of in silico analyses to RA-FLS-specific biological observations based on experimental evidence (Supplementary Table 2). For each module, we formulated prior knowledge based on small-scale experiments as observations and compared them with the results of the corresponding virtual experiment. Initial conditions were set to mimic the corresponding experimental settings. We used input propagation and identification of trap spaces to evaluate the phenotype-specific modules and the global model’s behaviour. First, we assessed the model’s behaviour by comparing the intended/ expected biological behaviour and the observed one after the process. In general, we faced some difficulties in assessing the models’ behaviour that can be summarised in the following categories: (a) not all components mentioned in the experimental settings included in the models, (b) experimental information not available for all components included in the models, (c) global behaviour consistent with experimental observations, but intermediate mechanisms only partially coherent. For some cases, further testing of the models revealed additional conditions needed to replicate the anticipated results. These additional “model conditions” that appeared to regulate the biological process are not experimentally proven (Supplementary Table 2). The perturbation experiments are also provided as knockout matrices for input conditions equal to 0 and equal to 1, respectively (Supplementary Tables 3 and 4).

#### Inflammation (Inflammation_phenotype in the models)

We formulated six biological scenarios to model Inflammation using the modules and the global RA-FLS model. TNF, one of the major pathways regulating Inflammation, was only able to activate the phenotype with the additional activation of the IKBA/NFKB/RELA complex. IKBA/NFKB/RELA complex is an input for activating the NFKB pathway, which is a key regulator of transcriptional responses to TNF^41–43^. IL-6 activation is a sufficient signal for activating Inflammation, as no additional inputs are needed to propagate the signal until the phenotype. Regarding IL-17, similar to TNF, the NFKB pathway is needed to activate the inflammation phenotype^43–45^.

#### Matrix degradation (Matrix_Degradation_phenotype in the models)

Cartilage destruction is one of the debilitating characteristics of RA. Activated RA-FLS have been shown to attach to the cartilage surface and release matrix-degrading enzymes. Matrix metalloproteinases (MMPs) play a pivotal role in cartilage destruction^46^. Our model reproduced the destructive role of two MMPs, MMP1 and MMP9, in activating the matrix degradation phenotype in both the phenotype-specific module and the global model.

#### Bone erosion (Bone_Erosion_phenotype in the models)

Bone erosion is another significant characteristic of RA. Synovitis, along with the production of proinflammatory mediators like Wnt (wingless-related MMTV integration site) and receptor activator of nuclear factor κB ligand (RANKL), results in the differentiation of bone-resorbing osteoclasts, thereby stimulating local bone resorption^47^. Secreted frizzled-related protein 5 (SFRP5), the primary upstream negative regulator of the WNT pathway when active, negatively regulates bone erosion^48^. Trap spaces showed that with both the module and the RA-FLS model, we could reproduce Wnt and RANKL (in the presence of SFRP5) biological behaviour^49–51^. Simulations of the models revealed a key role for SFRP5 in activating the bone erosion phenotype, as in its absence, the Wnt canonical pathway gets activated and further activates bone erosion.

#### Cell proliferation (Cell Growth/Survival/Proliferation_phenotype in the models)

Various growth factors like platelet-derived growth factor (PDGFA) and transforming growth factor beta 1 (TGFB1), and cytokines like TNF regulate the proliferation of RA-FLS^52,53^. Platelet-derived growth factor (PDGF) is an essential mitogen for fibroblasts, including RA-FLS^54,55^. PDGFA activated cell growth in both the phenotype-specific module and the global model. However, other pathways can still activate cell growth.

#### Apoptosis (Apoptosis_phenotype in the models)

The impaired apoptosis process of RA-FLS is responsible for synovial hyperplasia and joint destruction. Major pathways regulating apoptosis include extrinsic (FASLG and TNF) and intrinsic (mitochondrial pathway with BCL2 family proteins). Extrinsic pathways like FASLG and TNF contribute to the activation of apoptosis. The apoptosis module and the global model were able to reproduce this behaviour. AKT is another intracellular regulator of apoptosis (anti-apoptotic agent), which, when kept ON, protects RA-FLS against the apoptosis induced by FASL through inhibition of BID cleavage. However, this scenario was not reproducible, as discussed in the material and methods.

One of the reasons for the inconsistency was the presence of a direct inhibition (CAV1-negative regulator) to the apoptosis phenotype and the inferred logical formula that comprised OR gates between the activators and the absence of inhibitors. This direct inhibition reflects missing mechanistic details related to CAV1 regulating apoptosis. The RA map depicts this information using a negative influence directly at the phenotype glyph. While permitted by the CellDesigner tool^56^, the lack of mechanistic details leads to an inferred rule from the tool CaSQ that is not optimal, as the tool is designed to use reactions (SBGN-PD) and not influences (SBGN-AF) as input. Therefore, to reproduce the apoptotic-resistant nature of RA-FLS, we modified the logical formula changing the gates from OR to AND, keeping CAV1 as a dominant-negative regulator, in what we will denote the *modified global model*.

In our modified model, we need to have CAV1 set as active to act as a dominant inhibitor of apoptosis. This condition is justified and relevant from a biological point of view, as experiments have shown that MIR192—which acts as a CAV1 inhibitor in our model—suppresses cell proliferation and induces apoptosis in human RA-FLS by downregulating caveolin 1. Moreover, MIR192 appears to be downregulated in RA synovial tissues and restoring its expression restores the growth-suppressive effects on RA-FLS by targeting CAV1^57^. Therefore, the model condition of having CAV1 always active implies the absence of its inhibitor, MIR192, in the RA settings.

All analyses were performed for both versions of the RA-FLS global model, unmodified and modified, as seen in Supplementary Table 2, to ensure that the formula change did not have any impact on the model’s behaviour regarding the other four phenotypes. All biological scenarios in Supplementary Table 2 were simplified and compiled into tables with the anticipated outcomes associated with Boolean values 0 (OFF) and 1 (ON). Value propagation analysis and trap space identification were applied, and heatmaps were used to visualise the results for both the global model and its modified version. We could not find experimental values for all the model inputs (88 inputs) in the RA condition. Therefore we opted to go for either 0 (no severe inflammation) or 1 (fully activated system, resembling more to an entirely inflammatory condition) for the remaining model inputs that were not part of the biological scenarios tested. In Supplementary Fig. 2, we can see the behaviour of the global and modified model when all inputs are zero. All the phenotypes exhibit the same behaviour except for apoptosis. In the modified model, an additional condition of MIR192, a regulator of the negative inhibitor of apoptosis set as active, was required to reproduce two relevant scenarios.

**Fig. 3:**
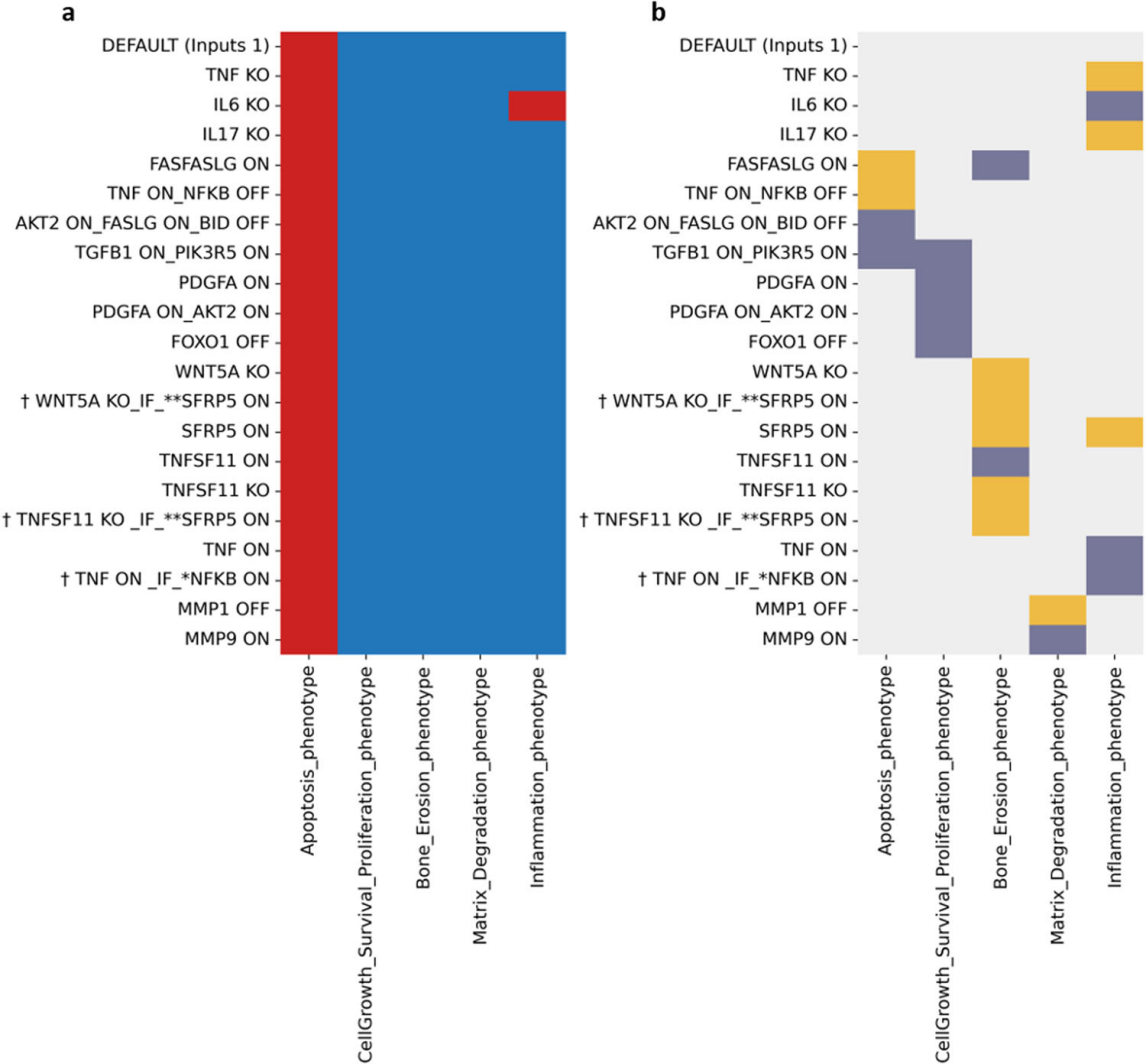
Trap spaces of the modified global model. Heatmap displaying the trap spaces for the tested biological scenarios in the modified global model (**a**) and the comparison between expected and obtained values shown with colour codes (**b**). The *y* axis shows all the tested scenarios’ names, as mentioned in Table 2, regarding all the five phenotypes as outcomes on the *x* axis with DEFAULT inputs set to one. ^**†**^Represent scenarios where additional conditions were given as a known biological behaviour* or as model conditions**. Trap spaces colour codes: -1 (unfixed) , 0 (OFF) , 1 (ON) . Expectation graph colour codes: Score: expected value, obtained value; 1: Yes [OFF, OFF & ON, ON] ; 0: No [ON, OFF & OFF, ON (conflict)] , **-**1: Undefined .

We further analysed the modified model with input conditions as 1 (Fig. 3a). Under these conditions, apoptosis remains OFF, and all other phenotypes remain ON, except for inflammation, which becomes inactive when IL-6 is OFF. Furthermore, when all inputs resemble an inflamed state in these phenotypic behaviours, many biological scenarios were still reproduced (as seen in Fig. 3b, represented with dark-grey colour). Lastly, some conflicts were observed between the expected and the displayed behaviour, represented in yellow. These conflicts were resolved with additional biological or model conditions in the next row.

In general, evaluating the models’ performance in regards to more mechanistic processes and intermediates that involved more complicated scenarios was not possible in several cases, as experimental information is currently missing, and we could not conclude on the coherence of these results. This is reflected in Fig. 3b (and also Supplementary Fig. 2B, D) for the cases represented with light grey colour. Nevertheless, these scenarios could serve as interesting hypotheses for further experimental testing.

### Calculating continuous time phenotypic probabilities

The RA-FLS model, in both versions, was used to calculate phenotypic probabilities. We used the software MaBoSS to reproduce representative scenarios with different parameters (discussed in “Materials and methods”)^58^. One scenario per phenotype from those listed in Supplementary Table 2 was selected for simulations, and results showed that in all cases but apoptosis, the two model versions were able to reproduce the experimental observations. In brief, we performed the following simulations:

#### Inflammation

**Fig. 4:**
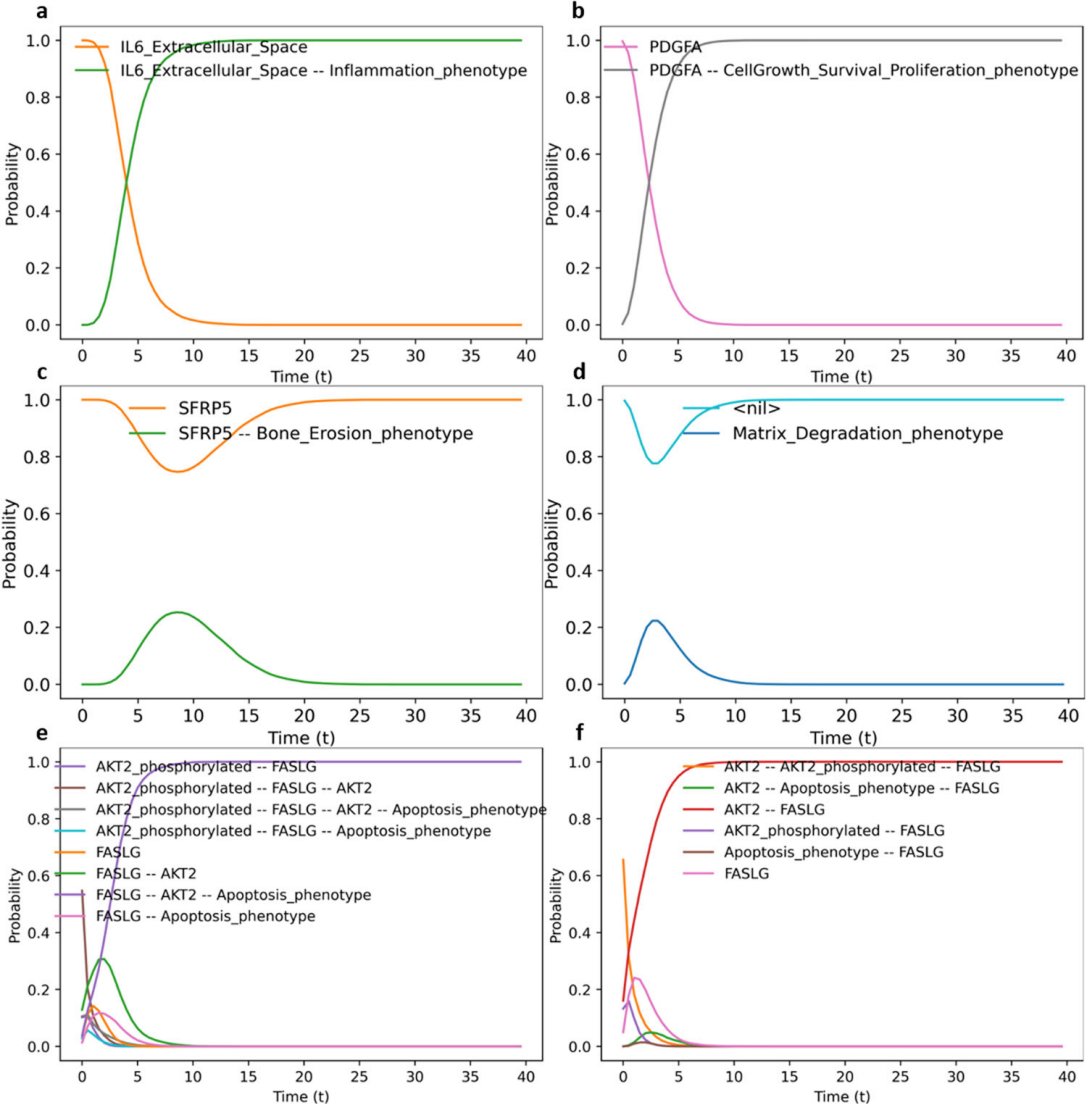
Calculating continuous time phenotypic probabilities of the selected initial conditions. **a** Simulation with IL6_Extracellular_space active and Inflammation as output. The Inflammation phenotype gets activated in the presence of IL-6. **b** Simulation with PDGFA active and Cell proliferation as output. The Cell proliferation phenotype gets activated in the presence of PDGFA. **c** Simulation with TNFSF11 (RANKL) inactive, SFRP5 active and Bone erosion as output. The Bone erosion gets deactivated in the presence of SFRP5 and the absence of TNFSF11. **d** Simulation with MMP1 inactive and Matrix degradation as output. Matrix degradation phenotype gets deactivated in the absence of MMP1. **e** Simulation with FASLG active, AKT2 active, BID inactive and Apoptosis as output (RA original global model). Apoptosis gets activated in the presence of FASLG, AKT2 and the absence of BID. **f** Simulation with FASLG active, AKT2 active, BID inactive and Apoptosis as output (RA modified global model). Apoptosis gets inactive in the presence of FASLG, AKT2 and the absence of BID due to the dominant-negative regulator CAV1.

IL-6, one of the main pathways regulating inflammation, was set active (1), and the Inflammation phenotype was chosen as the output. As a result, the simulation confirmed the activation of inflammation (Fig. 4a).

#### Cell proliferation

When PDGFA was set as active (1), cell proliferation was activated (Fig. 4b).

#### Bone erosion

When TNFSF11 (RANKL) was set as inactive (0) and the negative regulator SFRP5 as active (1), simulations showed the deactivation of bone erosion (Fig. 4c).

#### Matrix degradation

MMP1 was set as inactive by mutating its value as 0 (because it is an internal component and not an input to the model, its value must be fixed so that it cannot be changed by any upstream regulation, leaving it at 0 for the duration of the simulation) with matrix degradation chosen as the output. The simulation resulted in the deactivation of the matrix degradation (Fig. 4d).

#### Apoptosis

**Fig. 5:**
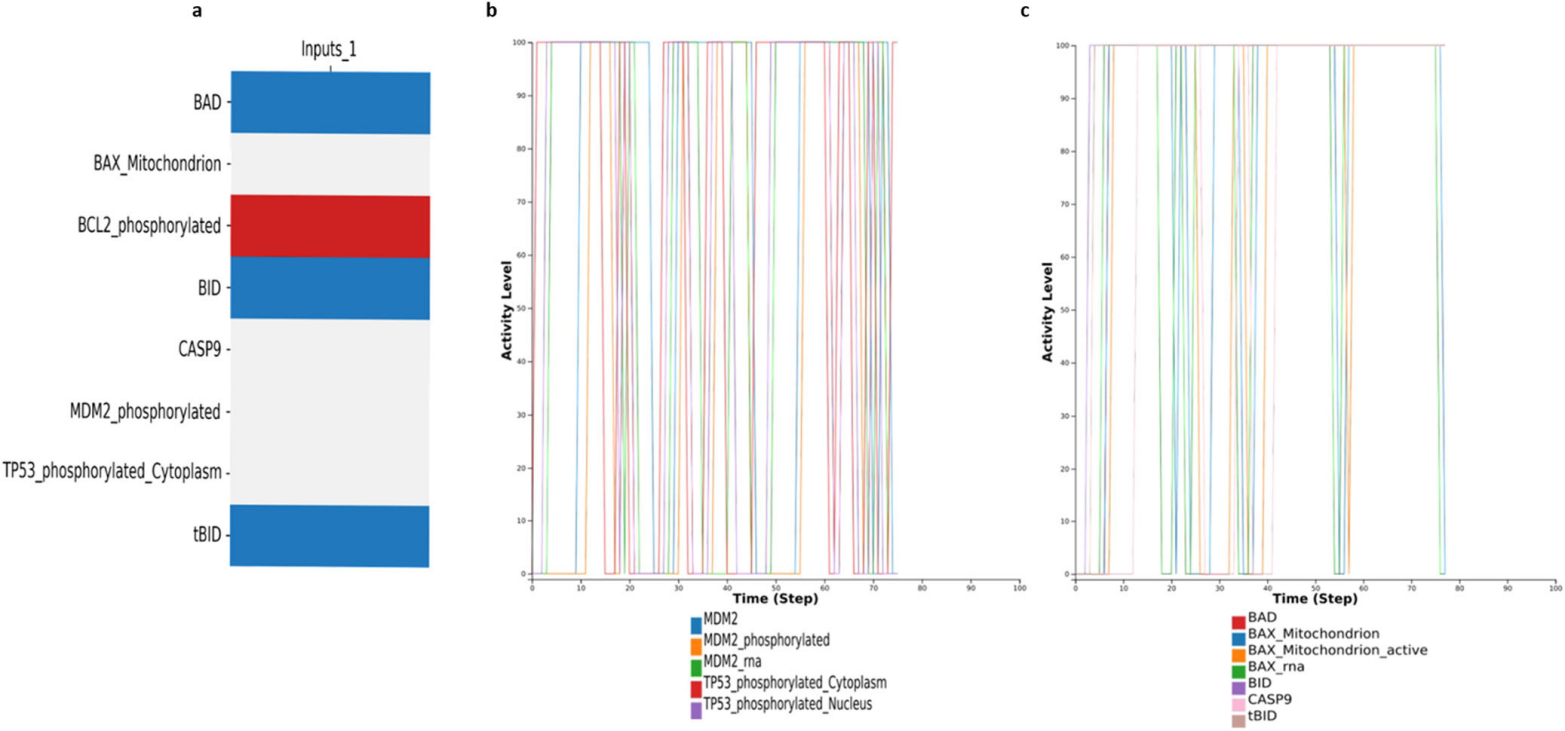
Oscillatory behaviour of TP53 and the mitochondrial proteins. **a** Trap spaces show that along with TP53 and MDM2, mitochondrial proteins like BAX and cytoplasmic protein CASP9 remain unfixed. **b** Simulation with CC shows TP53 and MDM2 oscillations while all inputs remain active. **c** Simulation with CC shows oscillatory behaviour of the mitochondrial proteins when all inputs remain active.

FASGL and AKT2 were set as active (1) and BID inactive (0). In the unmodified RA-FLS global model, apoptosis was found to be active in this condition, while in the modified global model, apoptosis was found to be inactive due to the dominance of the CAV1-negative regulator (as seen in Figs. 4e and 5f).

The results of the simulations are consistent with those from the trap space experiments for all scenarios tested.

### Reproducing known oscillatory behaviour of the P53-MDM2 interactions using the RA-FLS model

P53 is known to contribute to oscillatory behaviour through the P53 -MDM2 interactions. In short, a negative feedback loop on p53 is produced when p53 stimulates Mdm2 transcription, which in turn targets p53 for destruction. These P53 and MDM2 feedback-loop oscillations have been validated by mathematical and experimental models^59,60^ in various cell types and conditions. Moreover, TP53 directly takes part in the intrinsic apoptosis process by interacting with the multidomain members of the Bcl-2 family, causing mitochondrial outer membrane permeabilisation^61^. We wanted to see if the modified RA-FLS model could reproduce this dynamic behaviour. First, we calculated trap spaces when all inputs were active. The trap spaces analysis showed that the values of the proteins BAX, CASP9, TP53 and MDM2 are unfixed, while those of proteins BAD and BID are fixed in an active state, and protein BCL2 is fixed in an inactive state (Fig. 5a). The modified RA-FLS model was further analysed to study if the unfixed proteins exhibited an oscillatory behaviour under these conditions. We used the web-based modelling platform Cell Collective^62^ and asynchronously simulated the model setting all inputs as active. As seen in Fig. 5b, the model can reproduce the oscillations between TP53 and MDM2 entities and in Fig. 5c, we observe the oscillations in the intrinsic mitochondrial pathway of apoptosis.

### Reproducing mono and combined drug therapy in RA

Disease-modifying antirheumatic drugs (DMARDs), such as conventional synthetic (csDMARDs) (methotrexate, hydroxychloroquine), biologic (TNF-α inhibitors, IL-6 inhibitors), and targeted synthetic medications (tsDMARDs) (pan-JAK- and JAK1/2-inhibitors), have provided the most encouraging outcomes for the treatment of RA^63,64^. Many drugs targeting TNF and IL-6 are already established in clinical treatment^65^. While the symptoms of inflammation and pain could be relieved with the cocktails of these medications, 30–40% of the patients fail to fully respond to treatment and experience periods of disease remission and relapse^66^. In addition, the side effects caused by most medications and the financial cost limit their use after a specific dosage^64,67^. In Table 1, we provide a list of the mono and combined drug therapies used to treat RA. The RA-FLS model can mimic the effects of the drugs and predict the outcome of combined perturbations.

**Table 1:** Mono and combined drug therapy in RA.

Mono drug therapy	Targets
Tocilizumab, Sarilumab	IL-6^98^
Etanercept, Infliximab, Adalimumab, Golimumab, and Certolizumab Pegol	TNF^65,98^
Tofacitinib (RA-FLS), Baricitinib, Itacitinib	JAK^98,99^
Secukinumab	IL-17^98^
Andecaliximab, Celastrol (RA-FLS)	MMP9^98,100,101^
Imatinib	PDGF^16,102^
Methotrexate	IL1B, PDGF, NFKB^103,104^
Anakinra	IL1B^105^

Combined drug therapy	Targets
Methotrexate + Sarilumab (or Tocilizumab)	NFKB, PDGFA, IL1B targeted by Methotrexate + IL-6 targeted by Sarilumab^106,107^
Methotrexate + Cyclosporine	NFKB, PDGFA, IL1B targeted by Methotrexate + Calcineurin targeted by Cyclosporine^108,109^
Methotrexate + Azathioprine	NFKB, PDGFA, IL1B targeted by Methotrexate + RAC1 by Azathioprine^110,111^
Methotrexate + Hydroxychloroquine	NFKB, PDGFA, IL1B targeted by Methotrexate + TLRs targeted by hydroxychloroquine^112,113^
Methotrexate + Infliximab or Golimumab	NFKB, PDGFA, IL1B targeted by Methotrexate + TNF targeted by Infliximab/Golimumab^114^
TNF inhibitor + Abatacept	TNF + CD80^*^, CD86*, CD28 targeted by Abatecept^115,116^
TNF inhibitor + Anakinra	TNF + IL-1 targeted by Anakinra^115^
Abatacept + Anakinra	CD80*, CD86*, CD28 targeted by Abatecept + IL-1 targeted by Anakinra^115^

*Absent in the model.

**Fig. 6:**
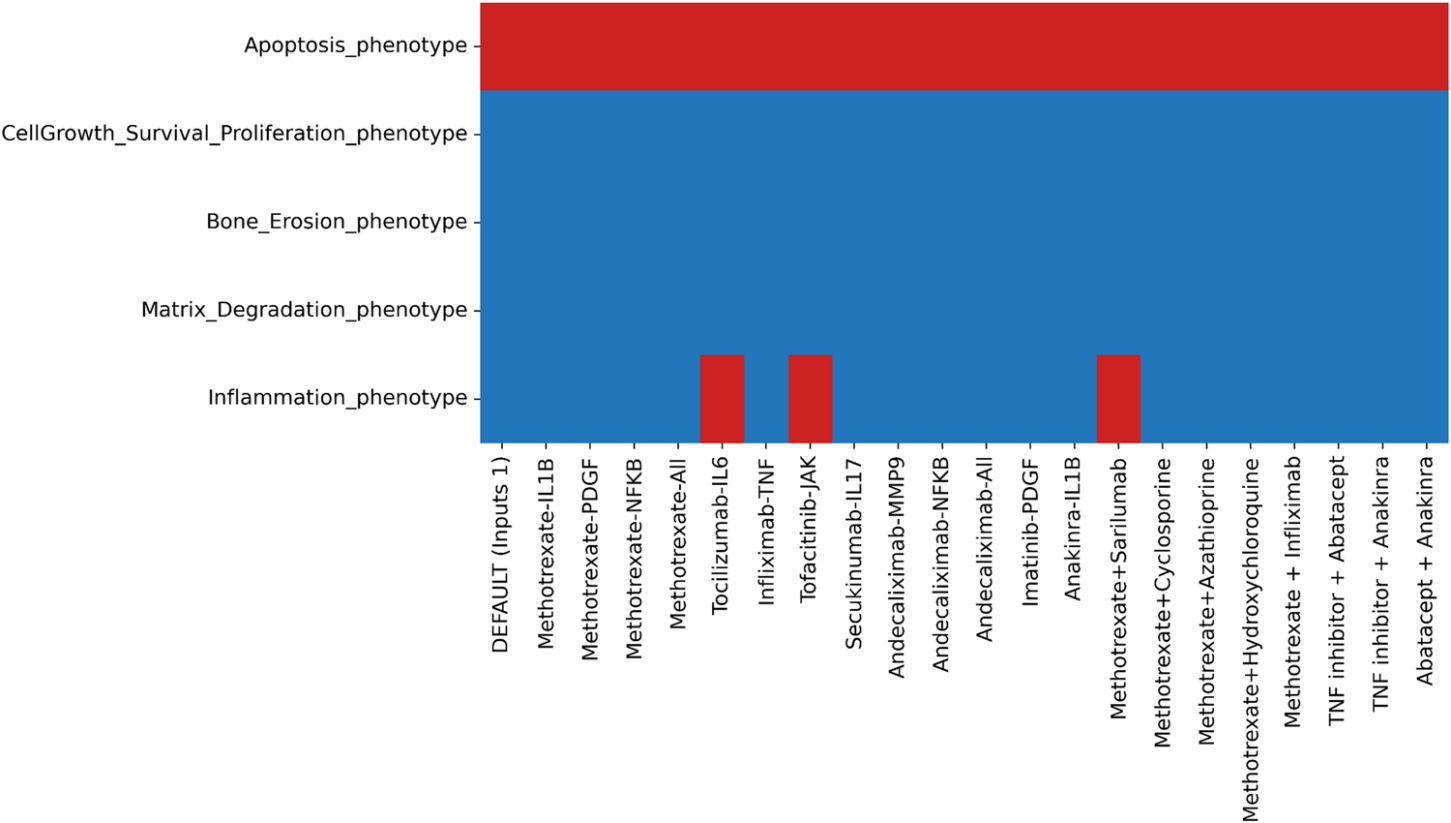
Heatmap showing identified trap spaces regarding the five phenotypes while testing different drug targets with DEFAULT inputs conditions as 1 with the modified version of the RA-FLS model.

Analysis was performed with the modified version of the RA-FLS model, DEFAULT input conditions as 1, as they are closer to the inflamed conditions in the RA joint. In addition, we set CAV1 as 1 to reproduce the apoptosis-resistant phenotype of the cells. Results shown in Fig. 6 confirm that IL-6, targeted by Sarilumab, Tocilizumab and JAK, targeted by Tofacitinib, Baricitinib and Itacitinib, were able to downregulate inflammation. Under these conditions, all other phenotypes, except apoptosis, were found to be active and not impacted by the drug treatment, implying that their regulation is more complex and requires additional targeting. We also tested drug combinations that can be administered to RA patients. The combination of Methotrexate and Sarilumab was seen to deactivate inflammation successfully. Our results corroborate the experimental findings of clinical trials that demonstrated better efficacy of the sarilumab targeting IL-6, either as monotherapy versus adalimumab, which targets TNF or in combination with csDMARDs versus placebo and csDMARDs^68^. Indeed, in our modelling framework, all results with anti-TNF drugs were inadequate to entirely suppress the inflammation phenotype, suggesting that blocking TNF alone is insufficient to hamper the signal. Our results also follow the findings of ref. ^69^, which demonstrated that IL-6 suppression was more successful in downregulating key transcription factors leading to inflammation in patients treated with anti-TNF treatment.

### Identification of novel targets and candidate drugs via drug repurposing analysis

The main objective of our modelling study was to find conditions that can induce apoptosis and downregulate cell proliferation, inflammation, matrix degradation and bone erosion in RA-FLS. This included identifying targets and available drugs that could act upon those targets. To proceed, we decided to focus on the direct upstream regulators of each phenotype, study their role based on the logical formula that describes their effect on the phenotype, and search for the available drugs in clinical trials and in vivo and in vitro studies. The available drugs targeting the upstream regulators of each phenotype can be seen in Supplementary Table 5.

In our model, one of the core conditions to reproduce RA-FLS behaviour is setting CAV1 as always active (being a negative regulator of apoptosis), implying a downregulation of its inhibitor, MIR192, which is also an input. This condition is sufficient to reproduce the RA-FLS resistance to apoptosis along with the activation of cell proliferation, inflammation, bone erosion and matrix degradation. We kept the same default model conditions (all inputs active and MIR192 inactive) for the drug-targeting simulations using the modified RA-FLS model and the Cell Collective simulation platform^62^ (Supplementary Fig. 3).

According to the logical formula, apoptosis is activated when CAV1 is inhibited and at least one activator is present. Interestingly, the negative regulator of apoptosis, CAV1, can be targeted by third-generation bisphosphonates (BPs), as demonstrated in various studies, although not always RA-relevant. Incadronate inhibits CAV1 expression in PC3 prostate cells^70^. Furthermore, studies on human epithelial fibroblasts show the negative impact of BPs on the expression of genes essential for their growth and differentiation at medium-low therapeutic doses in the long term^71,72^. As a substitute for patients who did not respond to conventional medication or who did not have easy access to biologics treatment, BPs have also been tested in RA patients. More specifically, scientists studied intravenous pamidronate administration for refractory rheumatoid arthritis. Results showed that pamidronate infusions were beneficial for most patients, but the alleviation of symptoms did not last for more than six months^73^. More recently, Zoledronic acid (ZA) was tested in combination with Methotrexate (MTX) in 66 RA patients for its efficacy in inhibiting RA disease activity. The combined treatment of ZA and MTX effectively reduced disease activity, fracture risk and bone pain in patients with RA-derived secondary osteoporosis^74^. A clinical trial involving 28 patients to assess the effects of ZA on patients with early-stage RA and low disease activity was concluded in 2018, but the results are not yet available (https://clinicaltrials.gov/ct2/show/NCT02123264). ZA has also been used to induce cell apoptosis in human and murine osteoclast precursors and mature osteoclast-like cells by triggering ROS- and GSK-3β-mediated Mcl-1 downregulation^75^. According to our model, BPs could also alleviate RA symptoms and inflammation, as they could inhibit CAV1 and activate apoptosis in RA-FLS. The inhibitory effects of ZA on tumour-related growth factor IL-6 in hormone-resistant prostate cancer cell lines have also been studied. The study showed the inhibitory effect of ZA on IL-6 secretion, which in turn resulted in increased apoptosis^76^. Keeping the default model conditions, we performed the simulations targeting CAV1 with Zoledronic acid. The results showed that when CAV1 is inhibited, apoptosis will become activated (Supplementary Fig. 4).

Inflammation in RA is one of the most characteristic symptoms. The inflammation phenotype in the RA-FLS model has thirteen direct upstream regulators. Among them, we find TNF, IL-6, IL17A, IL1B and NFKB, which represent common RA targets, and chemokines, such as CXCL8, CXCL9, CXCL10 and CXC11, two interferon proteins IFNB1, IFNA1, and lastly IRF1, IRF5 and IRF7. From all the direct upstream regulators, IRF1, 5 and 7 seem to control the fate of phenotype and are all regulated upstream by the IL-6 pathway. We performed simulations under the default conditions targeting IL-6, resulting in the complete inactivation of inflammation (Supplementary Fig. 5).

Bone erosion is directly dependent on the activation of eight upstream regulators. Besides TNF, IL17A and IL1B, known targets of RA therapy, we also find IL7, TNFRSF11, FOS, JUN and NFATC1 to exert direct control over the phenotype. We were able to find drugs that inhibit all five of them, with one having been in clinical trials for patients with multiple sclerosis (MS), but based on misrepresentation of preclinical data (GSK2618960 for IL7R). Results showed that simultaneous blocking of IL7 and AP-1 (JUN and FOS) leads to the inactivation of bone erosion under the default model conditions (Supplementary Fig. 6).

Matrix degradation in the RA-FLS model seems to be controlled exclusively by zinc-dependent endopeptidases, namely four MMPs and a disintegrin and metalloproteinase with thrombospondin motif (ADAMTS) member, ADAMTS4. Among these proteins, MMP3 is the one that exerts complete control over the phenotype for the given default conditions, and targeting only MMP3 was sufficient to inhibit matrix degradation (Supplementary Fig. 7).

Regarding cell proliferation, eight direct upstream regulators can affect the phenotype, namely TNF, NFKB, P38, BCL2, RPS6KB1, ADAMTS9, CREB1 and YWHAQ. TNF and NFKB are both usual targets of RA therapy. Regarding NFKB, Bortezomib is a proteasome inhibitor that has been shown to downregulate its pathway in cancer cells^77^. Bortezomib was also shown to attenuate murine collagen-induced arthritis^78^ and was recently found to improve the joint manifestations of rheumatoid arthritis in three patients^79^. P38 was once considered a promising target for developing new anti-inflammatory drugs to treat RA and other inflammatory diseases. However, the results in clinical trials were disappointing^80^. We identified drugs for two more upstream regulators, namely BCL2 and CREB1, and drugs that could target FOXO1, the upstream transcription factor of YWHAQ. In default conditions, among the eight regulators, CREB1 and YWHAQ were shown to inhibit completely the cell proliferation phenotype when targeted (Supplementary Fig. 8).

To recapitulate, the RA-FLS model was used to predict the best combinations for activating apoptosis and inhibiting inflammation, cell proliferation, bone erosion and matrix degradation. According to the model, the desired effect can be achieved by blocking CAV1, IL-6, IL7 and AP-1 (JUN and FOS), CREB1 and YWHAQ, and MMP3. For blocking CAV1, we have three potential drugs, Pamidronate, Incadronate and Zoledronic Acid (ZA), with the latter having been tested for RA (Supplementary Table 5). Regarding IL-6, Tocilizumab and Sarilumab are common biologics administered to RA patients (Table 1). IL7 activity can be targeted with GSK2618960, a monoclonal antibody against IL7R (Supplementary Table 5). Concerning AP-1, we identified Acitretin and T-5224. Acitretin, a retinoid, has been tested in clinical trials for various diseases. While Acitretin acts by binding and activating all nuclear subtypes of retinoid X receptors and retinoic acid receptors, it has been suggested that retinoic acid and its receptors regulate the differentiation and proliferation of epidermal keratinocytes by acting as an antagonist of activating protein-1 (AP-1). In psoriasis, it was also used in a small cohort of patients in combination with Etanercept (anti-TNF) to improve response to therapy, with encouraging results. T-5224, a selective inhibitor of c-Fos/AP-1, has been tested in a murine model of acute kidney injury (Supplementary Table 5). For CREB1, we could only identify one inhibitor, 666-15, while for YWHAQ, we identified AS1842856, which can target and inhibit FOXO1, an upstream regulator of YWHAQ. However, both inhibitors have only been tested in mice studies. Lastly, for MMP3, a variety of broad-spectrum MMP inhibitors are available. Some have already been tested in RA patients (like Cipemastat or Apratastat) but failed to produce significant results. Most drugs targeting MMPs, in general, and MMP3, in particular, have been tested primarily on cancer disease (Supplementary Table 5).

## Discussion

In this work, we focused mainly on the mechanisms regulating inflammation, matrix degradation, bone erosion, apoptosis, and cell proliferation, in RA-FLS. Our framework presents the experimental and clinical data and model results in an approachable manner which links various software applications and data. To build our large-scale model, we used the state-of-the-art RA map^19^ and the map-to-model framework proposed in ref. ^33^. Five-phenotype-specific modules and a global model were extracted and further enriched using RA-FLS-specific literature and single-cell data. The inferred models were further analysed using a variety of software, such as GINsim^81^, bioLQM^40^, MaBoSS^82^ and the CoLoMoTo notebook^35^. To our knowledge, the RA-FLS model is the first large-scale model that describes these cells’ signalling and gene regulation mechanisms in disease-specific settings.

Rheumatoid arthritis fibroblasts, are cells that possess complex traits, undergo phenotypic transformations and contribute to the sustained inflammation of the joints and the disease perpetuation. A simple phenomenological model cannot grasp the way multiple molecular pathways act in coordination to regulate this transformation. The debilitating symptoms of the disease include more than one phenotypic outcome, and one cannot claim to study sufficiently the mechanisms responsible for these outcomes by focusing only on a single subgroup of them. Especially so, when the aim is to propose novel targets, and probable repurposed drugs and combinations of them. While the challenge and the complexity are not negligible, Boolean formalism with its qualitative nature, can be used to efficiently scale up simulations, especially for converging signalling networks.

To evaluate the model’s behaviour, we mined experimental evidence based on cell-specific small-scale studies (knockouts and knock-ins) from the literature. We used value propagation^34,36^, trap spaces computation^83,84^, asynchronous simulations^62,85^ and continuous time Boolean stochastic modelling^82^ to analyse the models’ behaviour and evaluate the results. The employed methodologies not only reduced the amount of time needed to evaluate the behaviour of our large-scale models but also provided the means to comprehend intricate outcomes under specific circumstances.

The RA-FLS model has successfully reproduced various experimental observations impacting the five phenotypes of interest. However, inconsistencies were also identified. For example, while working on apoptosis, we observed the inability of the apoptosis phenotype to be inactive in the presence of a negative regulator. It highlighted the insufficient knowledge related to the biological processes involved in regulating the phenotype and the limitations of the automated model inference. To leverage the missing information, we modified the logical formula making the inhibitor dominant, a choice also justified by experimental evidence. The modified RA-FLS model reproduces many biological scenarios based on experimental evidence, at least in the input–output scale and for most of the phenotypes tested.

Furthermore, it successfully reproduces known oscillatory behaviour regarding TP53 and MDM2 along with the mitochondrial pathway proteins. The modules and the global model helped to understand the input and output (phenotype) relationships in a rather extensive network, along with the understanding of the regulatory processes at different biological scales. Considering it as a first step for understanding the RA-FLS behaviour in different sets of initial conditions, the model contributes toward a better understanding of the mechanisms that drive cell proliferation, inflammation, and resistance to apoptosis, bone erosion, and matrix degradation.

We used the RA-FLS model to perform mono and combined drug therapy simulations using drugs and combinations already administered to RA patients to understand better each drug’s different mechanisms of action and the combined effects on the cellular phenotypic outcomes. With all inputs active and mimicking the inflamed joint, IL-6 produces a stronger signal for inflammation, as TNF and IL-17 require the additional activation of NFkB to regulate inflammation. In addition, while different DMARDs have distinct modes of action, they interfere primarily with the main pathways of the inflammation cascade^86^. The activation of cell proliferation during drug testing and drug combinations shows that additional growth factors and signalling pathways are involved in controlling this process. As PDGFA is the only growth factor targeted in this instance, it seems insufficient to exert substantial control over the phenotype. Regarding bone erosion, the WNT non-canonical pathway signalling protein RAC12 (targeted by Methotrexate and Azathioprine) exerts partial control over the phenotype. However, bone erosion can also be triggered by other routes like RANKL (TNFSF11) and the WNT canonical pathway, thus keeping bone erosion active.

The next step was to identify possible targets that could enhance the desired effect of the treatment. Our goal was to achieve activation of apoptosis, suppression of inflammation, bone erosion, matrix degradation and cell proliferation for RA-FLS. To do so, we identified the direct regulators of the five different phenotypes and used drug repurposing analysis to identify drug candidates for these targets. More specifically, we used the logical formulae of the Boolean model to identify the direct regulators of each phenotype that could change the phenotypic state if targeted. We used several databases and dedicated sites to infer information about drugs and small molecules that target the action of these regulators. Some of these drugs had already been tested in RA or were administered in the context of other diseases. We had to tag discontinued drugs due to side effects, even when administered in a different disease context. For some drugs, information on toxicity or trial reports was limited. For example, CTS-1027, an MMP inhibitor, had been in phase II clinical trials by Conatus Pharmaceuticals (licensed from Roche) to treat Hepatitis C Virus (HCV) infection. However, this study was discontinued due to abnormalities and adverse events in a subset of clinical trial participants. This compound, originally discovered by Roche, showed positive results for osteoarthritis treatment, but no development report is available for this study (https://www.pharmacodia.com/yaodu/html/v1/chemicals/8651920f3ba2e5f8bcd3e58ba0b48584.html). Similarly, Marimastat, a broad MMP inhibitor, was discontinued in phase II trials, as it demonstrated drug toxicity linked to musculoskeletal pain and stiffness initially involving small peripheral joints of the hands^87,88^.

While our framework can be used to identify potential drugs and drug targets, we cannot assess toxicity or drug dosage but suggest new potentially interesting drug combinations to be tested experimentally. In Table 2, we recapitulate the identified drugs for the highlighted targets. Combining them could help activate apoptosis and downregulate inflammation, matrix degradation, bone erosion and cell proliferation in RA-FLS.

**Table 2:** List of drugs found to upregulate apoptosis and downregulate inflammation, bone erosion, matrix degradation and cell proliferation, targeting the identified biomarkers revealed by the RA-FLS model.

Identified drugs	Target components	Target phenotypes and expected effect
Pamidronate, Incadronate, and Zoledronic Acid	CAV1	Apoptosis
Sarilumab, Tocilizumab	IL-6	Inflammation
GSK2618960, and T-5224, Acitretin	IL7 AP-1	Bone erosion
Batimastat	MMP3	Matrix degradation
666-15 and AS1842856	CREB1 YWHAQ (FOXO1)	Cell proliferation

Our analyses demonstrated that besides input–output relationships, it is also necessary to understand and elucidate the underlying mechanisms that control the different phenotypes. Unit testing, wherein collections of tests specify the anticipated behaviour connected with distinct system modules, could be applied to verify local behaviour (see refs. ^34,36^). This could be of particular interest for understanding the complexity of the apoptosis mechanism in relation to the extrinsic and intrinsic (mitochondrial) pathways. Moreover, the resulting inconsistencies between prior knowledge and model behaviour could serve as a helpful indicator for re-evaluating the inferred logical formulae. The availability of more quality datasets could help to analyse the models under different sets of initial parameters and study their relation to the phenotypic outcomes. Furthermore, topological analysis of the model’s structural characteristics could reveal hubs and nodes with high centrality measures^89^, which could guide the combination of in silico knockouts (KOs) and knock-ins (KIs) for simulating the models.

Our approach supports using systems modelling in preclinical and clinical settings to provide insights into the possible outcomes of drug combinations. Further optimisation of the RA-FLS model using patient-specific omic data integrated with other clinical parameters could help towards personalised treatments.

## Materials and methods

### Generation of RA-FLS phenotype-specific modules and the global model

Analysing complex biological networks often requires a workflow that includes several different tools with different specifications and requires many additional steps to harmonise the whole procedure. The CoLoMoTo Interactive Notebook provides an integrated environment to execute, share, and reproduce analyses of qualitative models of biological networks^35^. The framework is available as a Docker image on Linux, MacOS and Microsoft Windows with the tools already installed and ready to be run with a Jupyter web interface executing the codes and visualising the results. The resulting notebook files can be shared and re-executed in the same environment. After installing the docker and accessing the latest colomoto-docker-V 2022-10-01 image, the notebook interface with Python modules for different tools was available to execute the analysis.

A global, comprehensive and fully annotated RA-specific map was published recently^19,90^. This map features components and interactions implicated in RA coming from various cell types. The RA-map CellDesigner XML file was first imported into the notebook using BioLQM and then converted into an executable RA-map model using CaSQ (CellDesigner as SBML-Qual), a tool for automated inference of large-scale, parameter-free Boolean models from molecular interaction maps based on network topology and semantics^33^. The executable RA model, in standard SBML-qual format^91^, can be further simulated and analysed using different modelling tools supporting the SBML-qual format. First, the RA model was sanitised using bioLQM to replace the model node Ids with node names. Then, the model was further used to extract five-phenotype-specific modules, apoptosis, cell proliferation, inflammation, matrix degradation, bone erosion, and a global model. The modules were extracted by selecting the phenotype and extracting its upstream part of the model through the connected components. Within this framework, we developed Python notebooks to validate RA-FLS-specific biological scenarios, drug testing and drug combination predictions.

### Model annotation and use of standards

Besides building and analysing large-scale biological models, one of the main challenges in systems modelling is the use of standards and proper annotation of such models to enhance a model’s reusability^92,93^. The models obtained in our pipeline are in a standard format (SBML-Qual) and thoroughly annotated using the MIRIAM scheme^94^, including PubMed IDs and pathway identifiers to annotate components and reactions. However, these elements are maintained and visualised only when the models are imported into the Cell Collective platform^62^ and lost when the models are further analysed with our CoLoMoTo notebooks due to the sanitisation process.

### Literature survey to find RA-FLS-specific biological scenarios

As our modelling efforts are based on RA-FLS, we did exhaustive literature mining to find biological scenarios to be tested for each phenotype with the individual modules and the global model. As a result, we formulated biological scenarios to be tested for each phenotype from the literature-5 for inflammation, 5 for bone erosion, 4 for cell proliferation, 2 for matrix degradation, and 4 for apoptosis phenotype (for more details, see Supplementary Table 2).

### Identification of trap spaces

We used *terminal trap spaces* to evaluate the asymptotic behaviour of our RA-FLS model. A *trap space* (also known as a stable motif or symbolic steady state) is a subspace from which the system cannot escape^83,84^. In particular, all possible successors of every state of a trap space belong to the same subspace. A trap space is *terminal* if it does not contain any smaller trap space. Terminal trap spaces accurately represent the Boolean model’s asymptotic behaviour and provide a good approximation of all attractors in practice.

Trap spaces can be efficiently identified using constraint-solving techniques without performing the simulation. Here we used the implementation provided by the bioLQM software for the identification of terminal trap spaces in reduced models obtained after input propagation, as explained below.

### Propagation of fixed components

Value propagation works on the propagation of assigned constant values to the corresponding downstream nodes. First, the cellular context is defined by assigning constant values to some model components, followed by a model reduction technique^34^. Then, for each fixed (constant) node, the corresponding value is inserted into the logical rule associated with each target node. If the rule can then be simplified to a constant, this new fixed value is further propagated into the logical rules of downstream nodes. This process is iterated until no further simplification can be made on the logical rules of the model^36^. Value propagation decreases the complexity of the model while preserving all attractors and terminal trap spaces.

### Continuous time stochastic simulations

In this type of simulation, continuous time phenotypic probabilities are being calculated. The probabilities associated with each phenotype represent the number of stochastic simulations leading to each phenotype from predefined initial conditions (based on experimental evidence that mimic the disease state).

MaBoSS is a C + + software for simulating continuous/discrete-time Markov processes applied to Boolean networks^58,82^. MaBoSS uses a specific language for associating transition rates with each node. Given some initial conditions, MaBoSS applies Monte-Carlo kinetic algorithm to the network to produce time trajectories and thus can associate probabilities with asymptotic solutions. The MaBoSS simulations were used to recreate at least one biological situation from each phenotype in the form of time-dependent (max time 40) trajectories using chosen parameters, more details of which could be found in the provided Python notebooks.

### Simulations using the web-based platform Cell Collective

We also used the modelling platform Cell Collective to simulate the global models^62^. Models in Cell Collective can be imported via the SBML-qual standard (CaSQ-produced SBML-qual model file was imported) or built from scratch. The Cell Collective SBML-qual import is compatible with model annotations and network layout. Furthermore, references kept in CellDesigner’s XML file’s MIRIAM section can be retrieved and seen in the Cell Collective platform.

### Modified RA model

While validating the biological scenarios in the original model, we observed that while Apoptosis should have been OFF, the phenotypic node was always active. The inability of the phenotype to turn OFF was further examined by studying the regulatory rules, which indicated a lack of mechanistic information on the biological process. CAV1 was part of the OR rule for the apoptosis regulators, as can be seen below:

**!CAV1_rna** | **CASP3_phosphorylated** | **CASP8** | **TNFRSF10A_rna** | **TNFRSF10B_rna** where **`|`** represents a disjunction and **`!`** is a negation.

The Boolean rule was modified appropriately to add CAV1 as a dominant regulator:

**!CAV1_rna&(CASP3_phosphorylated** | **CASP8** | **TNFRSF10A_rna** | **TNFRSF10B_rna**, where **`&`** represents a conjunction.

This rule change generated a modified global model where apoptosis remains OFF in the presence of a dominant-negative regulator corresponding to an actual RA condition where apoptosis remains deficient.

Five-phenotype-specific individual modules (apoptosis, cell proliferation, inflammation, matrix degradation and bone erosion) and the global model were then extracted from the modified RA model. Finally, all the biological scenarios were again tested with the modified individual modules and the five phenotypes global model and compared with the unmodified modules and model results.

### Drug-targeting (single, multiple targets) and drug combination analysis

We identified RA treatments that are administered regularly to RA patients and target different molecules, some of which are also RA-FLS specific (Table 2). We used both global models to simulate and analyse drug effects on the five selected phenotypes—Apoptosis, Cell proliferation, Inflammation, Matrix degradation and Bone erosion. We analysed the effect on the phenotypes for each drug by targeting its single and multiple targets in the global models. We also analysed the models by combining multiple drugs and their targets.

### Drug repurposing

We checked the logical formulae of all five phenotypes and extracted their upstream first-level regulators, as shown in Supplementary Table 5. For each regulator, we searched drugs with all clinical phases - launched, preclinical, phase 1, phase 2, and phase 3. For the latter, besides bibliographic search, the following sites were also used: https://clue.io/repurposing-app^95^; http://db.idrblab.net/ttd/^96^; https://go.drugbank.com/^97^. The results of this effort can be seen in Table 2, where the asterisk * denotes drugs already used in RA. We also thoroughly studied the mechanism of action of all the drugs (wherever information was available). We checked the default behaviour of the RA-FLS modified model concerning the five phenotypes by keeping all the inputs active and MIR192 inactive. MIR192 is input and needs to be OFF to activate the negative regulator of apoptosis CAV1, thus contributing to the state of RA pathology.

### Reporting summary

Further information on research design is available in the Nature Research Reporting Summary linked to this article.

## Supplementary information

Supplemental Material

Reporting Summary

## Data Availability

All data are available in the GitLab repository of the lab: https://gitlab.com/genhotel/rheumatoid-arthritis-large-scale-computational-modeling/-/tree/main.
