# Supplementary material for: A large-scale Boolean model of the rheumatoid arthritis fibroblast-like synoviocytes predicts drug synergies in the arthritic joint
Source: NPJ Syst Biol Appl. 2023 Jul 15;9:33. doi: 10.1038/s41540-023-00294-5 (PMC10349856; doi:10.1038/s41540-023-00294-5)
Supplement: Supplementary file 1 — Supplemental Material [file 41540_2023_294_MOESM1_ESM.pdf]

Supplementary material for

A large-scale Boolean model of the Rheumatoid Arthritis Fibroblast-Like Synoviocytes predicts drug synergies in the arthritic joint

Vidisha Singh[1], Aurelien Naldi[2], Sylvain Soliman[2], Anna Niarakis[* 1,2]
*corresponding author

[1] Université Paris-Saclay, Laboratoire Européen de Recherche pour la Polyarthrite rhumatoïde - Genhotel, Univ Evry, Evry, France.
[2] Lifeware Group, Inria, Saclay-île de France, 91120 Palaiseau, France

Supplementary Table 1. The global RA-FLS model comprises 261 nodes. In Table 1, we indicate the biological source of each component.

No	Global RA-FLS model components	Source (RA-FLS=Rheumatoid Arthritis-Fibroblast-Like Synoviocytes, NR=No Reference, PBMCs= Human Peripheral Blood Mononuclear cells, TH=T Helper cell)
1.	ADAMTS4	RA-FLS
2.	ADAMTS9	RA-FLS
3.	AKT2	RA-FLS
4.	APAF1	RA-FLS
5.	APC	NR
6.	Apoptosis	RA-FLS
7.	ARHGAP35	NR
8.	ARHGDIB (PBMC)	PBMCs
9.	ARHGEF1 (PBMC)	PBMCs

10.	ARHGEF2	RA-FLS
11.	AXIN	NR
12.	BAD	RA-FLS
13.	BAK1	RA-FLS
14.	BAX	RA-FLS
15.	BCL2	RA-FLS
16.	BCL2L1	RA-FLS
17.	BCL2L11	RA-FLS
18.	BID	RA-FLS
19.	Bone erosion	RA-FLS
20.	BRAF	RA-FLS
21.	CALCINEURIN	RA-FLS
22.	CALM1	RA-FLS
23.	CALM13	RA-FLS
24.	CAMK2A	RA-FLS
25.	CASP3	RA-FLS
26.	CASP8	RA-FLS
27.	CASP8AP2	PBMCs
28.	CASP9	RA-FLS

29.	CAV1	RA-FLS
30.	CD14	RA-FLS
31.	CD19	PBMCs
32.	CD28	Synovial tissue, blood, TH1
33.	CD3E	Synovial tissue, PBMCs, TH1
34.	CD72	Blood, synovial fluid
35.	Cell growth/Survival/Proliferation	RA-FLS
36.	CFLAR	RA-FLS
37.	cGAS	RA-FLS
38.	CK1A (CSNK1A1)	RA-FLS
39.	COMMD1	NR
40.	COMP	RA-FLS
41.	CREB1	RA-FLS
42.	CRKL	PBMCs, synovial tissue
43.	CSK	RA-FLS
44.	CTNNB	RA-FLS
45.	CTNNB1	RA-FLS
46.	CTNND1	Synovial tissue
47.	CXCL10	RA-FLS

48.	CXCL11	RA-FLS
49.	CXCL12	RA-FLS
50.	CXCL16	RA-FLS
51.	CXCL8	RA-FLS
52.	CXCL9	RA-FLS
53.	CXCR4	RA-FLS
54.	CXCR6	RA-FLS
55.	CYBA	RA-FLS
56.	CYBB	NR
57.	CYCS	RA-FLS
58.	DAXX	RA-FLS
59.	DKK1	RA-FLS
60.	DOCK2	Synovial tissue
61.	DUSP4	RA-FLS
62.	DVL1	RA-FLS
63.	DYNLRB1	RA-FLS
64.	EGF	RA-FLS
65.	EGFR	RA-FLS
66.	ETS1	RA-FLS

67.	FADD	RA-FLS
68.	FAS	RA-FLS
69.	FASLG	RA-FLS
70.	FGF1	RA-FLS
71.	FGFR1	RA-FLS
72.	FN1	RA-FLS
73.	FOS	RA-FLS
74.	FOXO1	RA-FLS
75.	FOXO3	RA-FLS
76.	FRIZZLED	PBMCs, synovial tissue
77.	FYN	Macrophages, synovial tissue
78.	GAB1	RA-FLS
79.	GAB2	RA-FLS
80.	GNAI3	PBMCs
81.	GNB1	PBMCs
82.	GRB2	RA-FLS
83.	GSK3b	RA-FLS
84.	HCST	PBMCs, synovial tissue
85.	ICAM1	RA-FLS

86.	ICAM2	RA-FLS
87.	ICAM3	RA-FLS
88.	IFNA1	NR
89.	IFNB1	RA-FLS
90.	IFNAR1	RA-FLS
91.	IFNAR2	Macrophages
92.	IGF1	RA-FLS
93.	IGF1R	RA-FLS
94.	IKBA	RA-FLS
95.	IKBKE	RA-FLS
96.	IKK	RA-FLS
97.	IL17A	RA-FLS
98.	IL17RA	RA-FLS
99.	IL18	RA-FLS
100	IL1A	RA-FLS
101	IL1B	RA-FLS
102	IL1R1	RA-FLS
103	IL6	RA-FLS
104	IL6R	RA-FLS

105	IL6ST	RA-FLS
106	IL7	RA-FLS
107	Inflammation	RA-FLS
108	ILK	PBMCs, TH1
109	IRAK1	RA-FLS
110	IRAK4	RA-FLS
111	IRF1	RA-FLS
112	IRF3	RA-FLS
113	IRF5	RA-FLS
114	IRF7	Chondrocytes, TH1, macrophages
115	IRF8	NR
116	IRF9	RA-FLS
117	ISGF3	RA-FLS
118	ITAM	Synovial tissue
119	ITBG2	RA-FLS
120	ITGAL	RA-FLS
121	ITGAV	RA-FLS
122	JAK1	RA-FLS
123	JAK2	RA-FLS

124	JAK3	RA-FLS
125	JUN	RA-FLS
126	KLRK1	PBMCs
127	LBP	Synovial tissue, synovial fluid
128	LCK	Synovial tissue
129	LCP2	Synovial tissue, PBMCs, TH1
130	LEFTY2	RA-FLS
131	LRP156	Synovial tissue
132	LTBP1	RA-FLS
133	LY96	PBMCs
134	MAP2K1	RA-FLS
135	MAP2K2	RA-FLS
136	MAP2K3	RA-FLS
137	MAP2K4	RA-FLS
138	MAP2K6	RA-FLS
139	MAP2K7	RA-FLS
140	MAP3K1	RA-FLS
141	MAP3K14	RA-FLS
142	MAP3K234	RA-FLS

143	MAP3K5	RA-FLS
144	MAP3K7	RA-FLS
145	MAP3K8	RA-FLS
146	MAP4K1	Synovial tissue
147	MAP4K4	RA-FLS
148	MAPK1	RA-FLS
149	MAPK14	RA-FLS
150	MAPK3	RA-FLS
151	MAPK7	Synovial tissue
152	MAPK8	RA-FLS
153	MAPK9	RA-FLS
154	MAPKAPK2	RA-FLS
155	Matrix degradation	RA-FLS
156	MCL1	RA-FLS
157	MDM2	RA-FLS
158	MICB	PBMCs
159	MMP1	RA-FLS
160	MMP13	RA-FLS
161	MMP3	RA-FLS

162	MMP9	RA-FLS
163	MTORC1	RA-FLS
164	MYD88	RA-FLS
165	NCF	RA-FLS
166	NECTIN1	Synovial tissue
167	NECTIN3	NR
168	NFAT5	RA-FLS
169	NFATC1	Synovial tissue
170	NFKB	RA-FLS
171	NFKB1	RA-FLS
172	NLK	Macrophages and blood
173	NRAS	RA-FLS
174	NRF1	Synovial tissue
175	OSCAR	Synovial tissue, blood
176	p38MAPK	RA-FLS
177	PDE3A	NR
178	PDGFA	RA-FLS
179	PDGFRA	RA-FLS
180	PIK3R5	RA-FLS

181	PLCG1	TH1
182	PLXNB1	RA-FLS
183	PMAIP1	RA-FLS
184	PP2A	RA-FLS
185	PPP3CB	Synovial tissue, TH1
186	PRKACG	PBMCs
187	PRKCD	Synovial tissue, macrophages
188	PRKCH	PBMCs
189	PRRT2	NR
190	PTEN	RA-FLS
191	PTK2B	RA-FLS
192	PTPN11	RA-FLS
193	PTPN6	Synovial tissue, macrophages
194	PTPRC	RA-FLS
195	RAB5A	RA-FLS
196	RAC12	RA-FLS
197	RAF1	RA-FLS
198	RANK	RA-FLS
199	RANKL	RA-FLS

200	RAP1B	RA-FLS
201	RAPGEF1	PBMCs, synovial fluid
202	RASGRP3	PBMCs, macrophages, synovial fluid
203	RELA	RA-FLS
204	RHOA	RA-FLS
205	RIPK1	PBMCs, TH1
206	RPS6KA1	Synovial tissue, PBMCs, TH1)
207	RPS6KA5	Synovial tissue
208	RPS6KB1	RA-FLS
209	SARM1	RA-FLS
210	SFRP5	RA-FLS
211	SH2D1A	RA-FLS
212	SHC2	RA-FLS
213	SHP2	RA-FLS
214	SOCS3	RA-FLS
215	SOS1	RA-FLS
216	SRC	RA-FLS
217	SRF	PBMCs
218	STAT1	RA-FLS

219	STAT2	RA-FLS
220	STAT3	RA-FLS
221	SYK	NR
222	TAB1	RA-FLS
223	TAB2	NR
224	TAK1	RA-FLS
225	TBK1	RA-FLS
226	LEF	Macrophages
227	TCF	RA-FLS
228	TGFB1	RA-FLS
229	TGFBR1	RA-FLS
230	TICAM1	RA-FLS
231	TICAM2	NR
232	TIRAP	RA-FLS
233	TLR2	RA-FLS
234	TLR4	RA-FLS
235	TLR5	RA-FLS
236	TNF	RA-FLS
237	TNFAIP3	Synovial tissue, PBMCs, macrophages

238	TNFRSF10A	RA-FLS
239	TNFRSF10B	RA-FLS
240	TNFRSF1A	RA-FLS
241	TNFRSF1B	RA-FLS
242	TNFSF11	RA-FLS
243	TP53	RA-FLS
244	TP73	RA-FLS
245	TRADD	PBMCs, macrophages
246	TRAF2	RA-FLS
247	TRAF3	RA-FLS
248	TRAF3IP2	RA-FLS
249	TRAF5	RA-FLS
250	TRAF6	RA-FLS
251	TXK	RA-FLS
252	TYK2	Blood
253	TYROBP	Synovial tissue
254	VAV1	Synovial tissue
255	VAV2	Synovial tissue
256	WNT	Synovial tissue

257	WNT5A	RA-FLS
258	YWHAQ	RA-FLS
259	YY1	RA-FLS
260	ZAP70	PBMCs, TH1)
261	ZC3H12A	RA-FLS

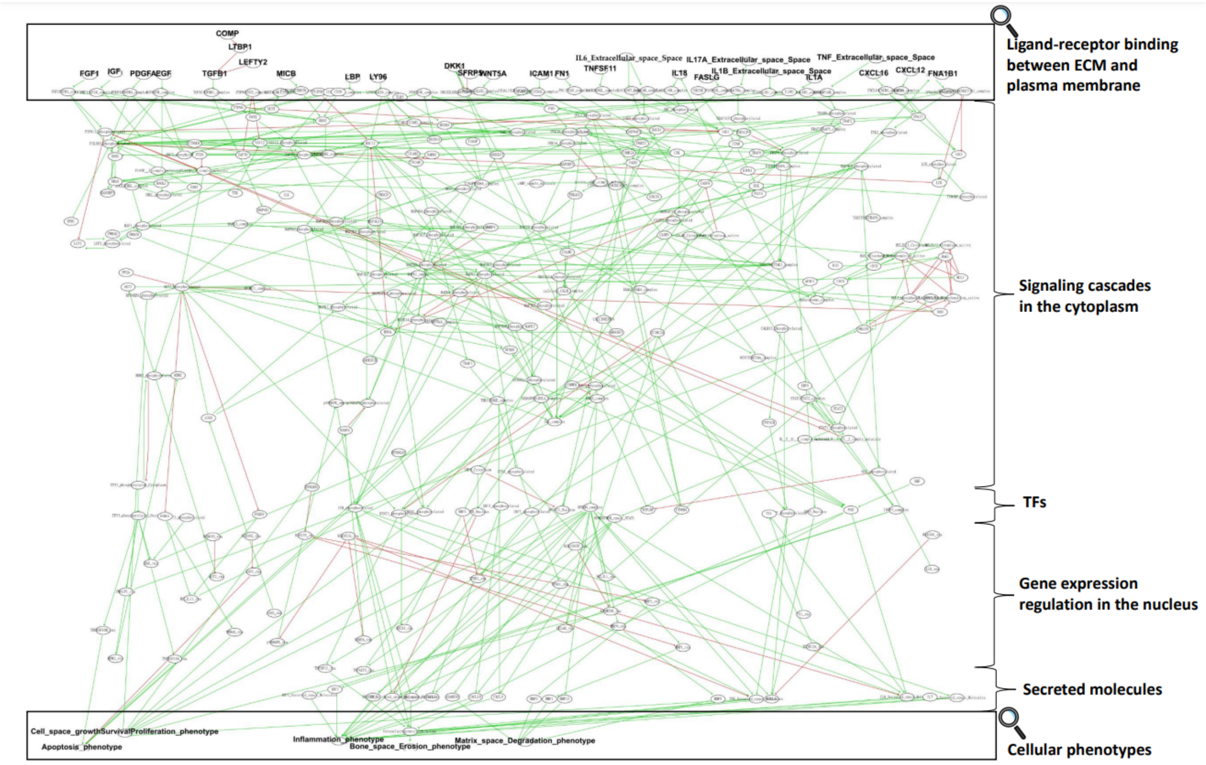

Supplementary Figure 1: The regulatory graph of the RA-FLS global model. The layout is similar to the molecular map, with ligands/receptors and proximal signalling at the top, second messengers in the cytoplasm, gene regulation in the nucleus, secreted components and phenotypic outcomes at the bottom of the graph. Green arcs denote activation, and blunt red arcs denote inhibition. The regulatory graph comprised 321 nodes and 515 interactions (magnifying glass vector from vecteezy.com).

Supplementary Table 2. Biological scenarios tested using the unmodified and modified RA-FLS global models and the phenotype-specific modules.

† displays RA-FLS specific scenarios reproduced with additional conditions of (* known biological behaviour, ** model conditions added to reproduce the RA-FLS biological scenarios. The added

model conditions are not experimentally proven).

Phenotypes	Biological scenarios	References	Experimental techniques/ origin	Validation using the phenotype-specific module	Validation using the RA-FLS global model	Validation using the modified global RA-FLS model
**Inflammation**	1) Anti-TNF-α monoclonal antibody results in a decrease in synovial inflammation **TNF KO**	Matsuno et al., 2002	SCID mice, RA synovium	Yes	Yes	Yes
	2) Synovial inflammation was significantly exacerbated by TNF-α administration **TNF ON**	Matsuno et al., 2002	SCID mice, RA synovium	Yes (by manually activating NFKB, which is a *known biological condition regulating TNF)	Yes (by manually activating NFKB, which is a *known biological condition regulating TNF	Yes (by manually activating NFKB, which is a *known biological condition regulating TNF
	3) Interleukin 6 knockout mice are resistant to antigen-induced experimental arthritis **IL6 KO**	Boe et al., 1999	IL6 KO mice	Yes	Yes	Yes
	4) Anti-IL17A antibody reduces joint inflammation in collagen-induced arthritis (CIA) **IL17 KO**	Lubberts et al., 2004; Nakae et al.,2003	CIA mice	Yes	Yes	Yes
	5) Secreted frizzled-related protein 5 suppresses inflammatory response in rheumatoid arthritis fibroblast-like synoviocytes **SFRP5 ON**	Kwon et al., 2014	RA-FLS human, ELISA	Yes	Yes	Yes

**Bone erosion**	1) Secreted frizzled-related protein 5 suppresses inflammatory response in rheumatoid arthritis fibroblast-like synoviocytes **SFRP5 ON**	Kwon et al., 2014	RA-FLS human, ELISA	Yes	Yes	Yes
	2) RANKL (TNFSF11) expressed on synovial fibroblasts is involved in rheumatoid bone destruction by inducing osteoclastogenesis **TNFSF11 ON**	Takayanagi et al., 2000	RA-FLS human, Northern blot	Yes	Yes	Yes
	3) RANKL (TNFSF11) knockout mice are protected from bone erosion in a serum transfer model of arthritis **TNFSF11 KO**	Pettit et al., 2001	TNFSF11 KO mice	Yes (by manually activating SFRP5, a **model condition)	Yes (by manually activating SFRP5, a **model condition)	Yes (by manually activating SFRP5, a **model condition)
	4) Wnt5a cKO mice were resistant to arthritis development **WNT5A KO**	MacLauchlan et al., 2017	Wnt5a KO mice	Yes (by manually activating SFRP5, a **model condition)	Yes (by manually activating SFRP5, a **model condition)	Yes (by manually activating SFRP5, a **model condition)
	5) Fas receptor induces apoptosis of synovial bone and cartilage progenitor populations and promotes bone loss in antigen-induced arthritis	Li et al., 2017; Lazić Mosler et al., 2019	Wild type mice and Fas KO mice	Yes	Yes	Yes

	**FASFASLG ON**					
**Cell proliferation**	1) PDGF stimulates the proliferation of synovial fibroblasts **PDGFA ON**	Sandler et al., 2006; Kameda et al., 2006	Human RA FLS, Western blot, BrdU assay, ELISA	Yes	Yes	Yes
	2) PDGF stimulates the growth of synovial fibroblast via interference with the Akt signalling pathway **PDGFA ON_AKT2 ON**	Terabe et al., 2009	Human RA FLS, Western blot	Yes	Yes	Yes
	3) Downregulation of FoxO1 is required to promote the survival of fibroblast-like synoviocytes in rheumatoid arthritis **FOXO1 OFF**	Grabiec et al., 2015	ELISA, immunoblotting and quantitative PCR	Yes	Yes	Yes
	4) TGFbeta exerts its growth and antiapoptotic effects on fibroblasts by activation of the PI3Kinase-AKT pathway **TGFB1 ON_PIK3R5 ON**	Kim G et al., 2002	RA-FLS human, chemical inhibition, Western blot	Yes	Yes	Yes
**Matrix degradation**	1) Inhibition of MMP1 alone results in a significant reduction of cartilage invasion by RASFs	Rutkauskaite et al., 2004	Quantitative PCR, ELISA, SCID mouse	Yes	Yes	Yes

	**MMP1 OFF**					
	2) MMP-9 stimulates RA synovial fibroblast-mediated inflammation and degradation of cartilage **MMP9 ON**	Xue et al., 2014	BrdU assays, ELISA, western blot and zymography	Yes	Yes	Yes
**Apoptosis**	1) Fas receptor induces apoptosis of synovial bone and cartilage progenitor populations and promotes bone loss in antigen-induced arthritis **FASFASLG ON -** original model, **FASFASLG ON_MIR192 ON (modified)** - modified_model	Lazić Mosler et al., 2019	Wild type mice and Fas KO mice	Original module - Yes, Modified module - Yes (by manually activating MIR192_rna to deactivate the dominant CAV1 negative regulator, a **model condition)	Yes	Yes (by manually activating MIR192_rna to deactivate the dominant CAV1 negative regulator, a **model condition)
	2) TNFα can induce apoptosis in RA-FLS when NF-κB is inhibited **TNF ON_NFKB OFF-** original model, **TNF ON_NFKB OFF_MIR192 ON (modified)** -modified model	Zhang et al., 2000	SCID mice	Original module - Yes, Modified module - Yes (by manually activating MIR192_rna to deactivate the dominant CAV1 negative regulator, a **model condition)	Yes	Yes (by manually activating MIR192_rna to deactivate the dominant CAV1 negative regulator, a **model condition)
	3) In RA-FLS, phosphorylation of AKT protects against Fas-induced apoptosis through	García et al., 2010	RA-FLS human, ELISA, Western blot	Original module - No, Modified module - Yes	No	Yes

	inhibition of Bid cleavage. **AKT2 ON_FASLG ON_BID OFF**					
	4) TGFbeta exerts its growth and antiapoptotic effects on fibroblasts by activation of the PI3Kinase-AKT pathway **TGFB1 ON_PIK3R5 ON**	Kim G et al., 2002	RA-FLS human, chemical inhibition, Western blot	Original module - unfixed, Modified module - Yes	Unfixed	Yes

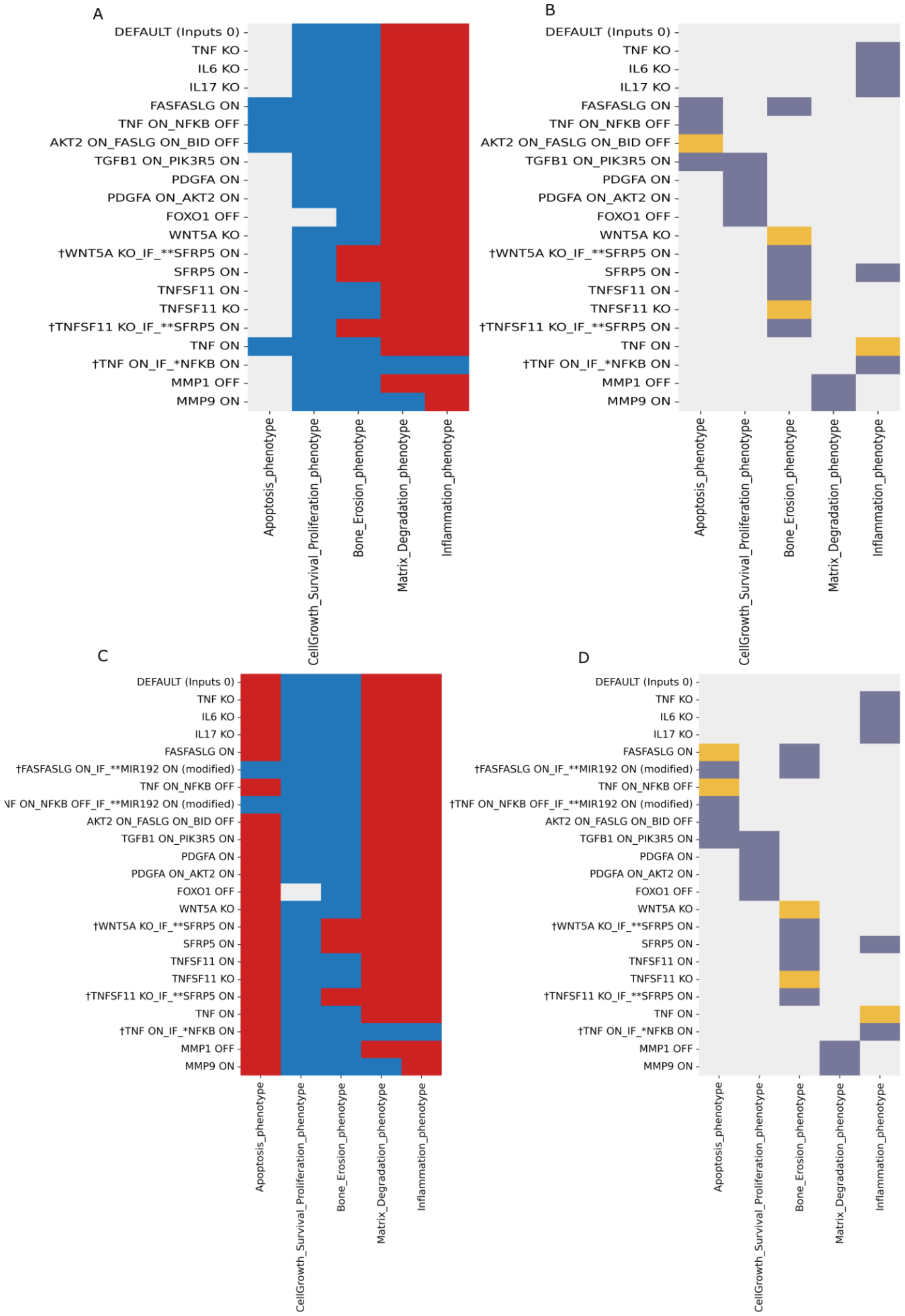

Supplementary Figure 2. Heatmap displaying the trap spaces for the tested biological scenarios in the unmodified global model (A), modified global model (C) and the comparison between expected and obtained values shown with colour codes for the unmodified (B) and the modified (D) models with DEFAULT inputs set to zero. The y-axis shows all the tested scenarios' names, as mentioned in table

2, in regard to all the five phenotypes as outcomes on the X-axis. † represent scenarios where additional conditions were given as a known biological behaviour* or as model conditions**.

Trap spaces colour codes: -1 (unfixed), 0 (OFF), 1 (ON)
Expectation graph colour codes:

Score: expected value, obtained value; 1: Yes [OFF, OFF & ON, ON]; **0**: No [ON, OFF & OFF, ON (conflict)], **-1**: Undefined

Supplementary Tables 3 and 4 can be found at the end of the document in a landscape orientation.

Supplementary Table 5. Direct upstream regulators of the five phenotypes and available drugs that target them

* drugs used in RA

Phenotypes	Logical formulae	Direct upstream regulators	Drugs targeting direct upstream regulators	No of clinical trials for the drug	In clinical trials/*in vitro* /*in vivo*
Apoptosis	!CAV1_rna&(CASP3_phosphorylated|CASP8|TNFRSF10A_rna|TNFRSF10B_rna	CAV1<br>CASP3<br>CASP8<br>TNFRSF10A<br>TNFRSF10B	**CAV1: Pamidronate**	**56**	**Pamidronate**<br>Phase1/2/3/4 myeloma, osteoporosis, arthroplasty, osteopenia, cancers (https://clinicaltrials.gov/ct2/results?cond=&term=Pamidronate&cntry=&state=&city=&dist=)
			**Zoledronic acid***	**380**	**Zoledronic acid***<br>Phase1/2/3/4<br>RA, osteoporosis, osteopenia, cancers, Parkinson's Disease, multiple myeloma, (https://clinicaltrials.gov/ct2/results?term=Zoledronic+acid&age_v=&gndr=&type=&rslt=&Search=Apply)
			**Incadronate**		**Incadronate**<br>RA (Zhao et al. 2006)<br>prostate cancer (Iguchi et al. 2006)

Cell proliferation	NFKB/N_complex|YWHAQ_rna|CREB1_phosphorylated|BCL2_phosphorylated|RPS6KB1_phosphorylated|TNF_Secreted_space_Molecules|ADAMTS9_rna|p38MAPK_phosphorylated	TNF<br>NFKB_complex<br>CREB1<br>BCL2<br>RPS6KB1<br>ADAMTS9<br>P38MAPK<br>YWHAQ**	**NFKB: Bortezomib***<br><br>**Salvianolic acid B*** (SA-B)<br><br>**P38MAPK: CDD-450*** ,<br><br>**SD-0006**<br><br>**R-1487 ***<br><br>**BMS-582949**<br><br>**Dilmapimod***<br><br>**Doramapimod***	**1040**<br><br>**2**<br><br>**16**<br><br>**4**<br><br>**10**<br><br>**11**	**Bortezomib**<br>Phase2/3/4<br>RA, multiple myeloma, lymphoma, leukaemia, different cancers<br>(https://clinicaltrials.gov/ct2/results?cond=&term=Bortezomib&cntry=&state=&city=&dist=), (Chen et al. 2011)<br><br>Rat model (Xia et al. 2018)<br><br>**CDD-450**<br>Phase 2<br>RA, psoriatic arthritis<br>**(**https://clinicaltrials.gov/ct2/results?cond=&term=CDD-450&cntry=&state=&city=&dist=**),**<br>(https://doi.org/10.1038/s41584-018-0003-y)<br><br>**SD-0006**<br>Cancers, type 2 diabetes, Alzheimer's Disease<br>Phase2/3/4<br>**(**https://clinicaltrials.gov/ct2/results?term=SD+0006&rank=1#rowId**0)**<br><br>Phase 1 (Goldstein et al. 2011)<br><br>**BMS-582949**<br>Phase 1/2<br>RA, psoriasis, atherosclerosis<br>(https://clinicaltrials.gov/ct2/results?cond=&term=BMS-582949&cntry=&state=&city=&dist=)<br><br>**Dilmapimod**<br>phase 1/2 RA, pulmonary Disease<br>(https://clinicaltrials.gov/ct2/results?term=Dilmapimod&draw=2&rank=2#rowId1)<br>**Discontinued** (Lakshmi et al. 2017)<br><br>**Doramapimod**<br>Phase 2<br>RA, psoriasis, Crohn's Disease<br>(https://clinicaltrials.gov/ct2/res

					ults?term=Doramapimod&draw=2&rank=2#rowId1)
			**Losmapimod**	**15**	**Losmapimod**<br>Phase2, Facioscapulohumeral Muscular Dystrophy (FSHD), glomerulosclerosis, focal segmental, pulmonary disease, chronic obstructive, acute coronary syndrome, atherosclerosis (https://clinicaltrials.gov/ct2/results?cond=&term=Losmapimod&cntry=&state=&city=&dist=)
			**LY2228820**	**5**	**LY2228820**<br>Phase 1, metastatic breast cancer, adult glioblastoma, epithelial ovarian cancer, advanced cancer (https://clinicaltrials.gov/ct2/results?cond=&term=LY2228820&cntry=&state=&city=&dist=)
			**PH-797804 ***	**16**	**PH-797804**<br>Phase 2, RA, chronic obstructive pulmonary disease, pulmonary disease, chronic obstructive, osteoarthritis, neuralgia, postherpetic neuralgia (https://clinicaltrials.gov/ct2/results?cond=&term=PH-797804&cntry=&state=&city=&dist=)
			**VX-745 ***	**5**	**VX-745**<br>Phase 2, Alzheimer's Disease, Huntington's Disease (https://clinicaltrials.gov/ct2/results?cond=&term=VX-745&cntry=&state=&city=&dist=), RA(Haddad 2001)
			**BCL2: Obatoclax**	**20**	**Obatoclax**<br>Phase 1/2 of different leukaemia and lymphoma (https://clinicaltrials.gov/ct2/results?cond=&term=Obatoclax&cntry=&state=&city=&dist=), (Or et al. 2020)
			**CREB1: 666-15**		**666-15**<br>*In vivo* mice studies (Xie et al. 2019; Li et al. 2016)

			**YWHAQ** targeted by upstream TF **FOXO1: AS1842856**		**AS1842856** (Zou et al. 2014), *In vivo* mice study (Nagashima et al. 2010), review (Calissi et al. 2021)
Inflammation	CXCL11|IRF7|CXCL8|IRF1|IFNB1_rna|IL1B_Secreted_space_Molecules|CXCL9|TNF_Secreted_space_Molecules|IL6_Secreted_space_Molecules|NFKB/N_complex|IL17A_Secreted_space_Molecules|CXCL10|IFNA1_rna|IRF5_Secreted_space_Molecules	TNF<br>IL6<br>IL17A<br>IL1B<br>NFKB<br>CXC11<br>IRF7<br>CXCL8<br>IFNB1<br>CXCL9<br>CXCL10<br>IFNA1<br>IRF5<br>IRF1	**CXCL8: Carboxyamidotriazole**<br><br>**Rivanicline**<br><br>**CXCL10: BMS-936557**<br><br>**CXCL11:** targeting by upstream IRF3 **Piceatannol**<br><br>**ADU-S100**<br><br>**IFNA1: Interferon alfa-n3**<br><br>**IFNB1: PEGylated IFN beta 1-a**	**10**<br><br><br><br>**2**<br><br>**1**<br><br>**3**<br><br>**11**<br><br>**15**	**Carboxyamidotriazole** Phase1/2 of different cancers (https://clinicaltrials.gov/ct2/results?cond=&term=Carboxyamidotriazole&cntry=&state=&city=&dist=)<br><br>*In vitro* study (Spoettl et al. 2007)<br><br>**BMS-936557** Phase2 colitis, ulcerative (Mayer et al. 2014), Crohn's Disease (https://clinicaltrials.gov/ct2/results?term=BMS-936557&draw=2&rank=1#rowId0)<br><br>**Piceatannol** Preclinical (https://clinicaltrials.gov/ct2/show/NCT04983017?term=Piceatannol&draw=2&rank=1), In vivo mice study (Dang et al. 2004)<br><br>**ADU-S100** Phase1/2 of cancer/lymphoma (https://clinicaltrials.gov/ct2/results?cond=&term=ADU-S100&cntry=&state=&city=&dist=)<br><br>**Interferon alfa-n3** (https://go.drugbank.com/drugs/DB05258), Phase1/2 HIV infections, different cancer/lymphoma/myeloma (https://clinicaltrials.gov/ct2/results?cond=&term=Interferon+alfa-n3&cntry=&state=&city=&dist=)<br><br>**PEGylated IFN beta 1-a** Phase3 multiple sclerosis, SARS-CoV Infection

					(https://clinicaltrials.gov/ct2/results?cond=&term=PEGylated+IFN+beta+1-a&cntry=&state=&city=&dist=), (Calabresi et al. 2014)
Bone erosion	Osteoclastogenesis - IL17A_Secreted_space_Molecules|TNFSF11_rna|IL1B_Secreted_space_Molecules|TNF_Secreted_space_Molecules|FOS|JUN_phosphorylated|IL7|NFATC1_Secreted_space_Molecules	TNF<br>IL17A<br>IL1B<br>IL7<br>TNFRSF11<br>FOS<br>JUN<br>NFATC1	**JUN, FOS (AP-1)**<br>**T-5224,**<br><br>**Acitretin**<br><br>**IL7**<br>**GSK2618960 (anti-IL7R)**<br><br>**TNFRSF11***<br>**Denosumab**<br><br>**NFATC1**	**45**<br><br>**3**<br><br>**81**	**T-5224**<br>*In vivo* mice study (Ishida et al. 2015)<br><br>**Acitretin**<br>**https://www.ncbi.nlm.nih.gov/books/NBK519571/**<br>Phase4 psoriasis, Phase1/2 melanoma, HIV infections, Alzheimer's Disease (https://clinicaltrials.gov/ct2/results?cond=&term=acitretin&cntry=&state=&city=&dist=)<br>https://www.ncbi.nlm.nih.gov/pmc/articles/PMC3219164/<br><br>**GSK2618960**<br>Multiple sclerosis<br>**(https://clinicaltrials.gov/ct2/show/NCT01808482)**<br><br>**Denosumab**<br>MOA (Bruhn 2010), Phase2/3/4 RA, osteoporosis, cystic fibrosis, bone resorption, breast cancer, cancers (https://clinicaltrials.gov/ct2/results?term=Denosumab&age_v=&gndr=&type=&rslt=&phase=1&Search=Apply)<br><br>**INCA-6**<br>*In vitro* and cells (Roehrl et al. 2004), Preclinical
Matrix degradation	MMP13|MMP9|MMP1|ADAMTS4|MMP3	MMP13<br>MMP9<br>MMP1<br>MMP3<br>ADAMTS4	MMP9, MMP3, MMP13,MMP1<br>**CTS-1027**	**4**	**CTS-1027**<br>**Hepatitis**<br>(https://clinicaltrials.gov/ct2/show/NCT00925990), https://drugs.ncats.io/drug/2QD3F58224<br>**It was discontinued due to laboratory abnormalities and**

					**adverse events in a subset of clinical trial participants.**
			**Marimastat**	**4**	**Marimastat**<br>Glioblastoma, breast, ovarian and small and non-small cell lung cancer (https://clinicaltrials.gov/ct2/results?cond=&term=Marimastat+&cntry=&state=&city=&dist=)<br>**It was discontinued as it failed to show superior efficacy over either standard chemotherapy or placebo.**
			**Cipemastat***	**13**	**Cipemastat**<br>Inhibits MMP-1, MMP-3 and MMP-9 but did not prevent the progression of joint damage in RA **(**Vandenbroucke, R., Libert, C. 2014)<br>**It was discontinued as it failed to improve radiographic scores**
			**Apratastat (TMI-005)** *	**1**	**Apratastat**<br>TACE and MMPs inhibitor (Thabet and Huizinga 2006)<br>**It was discontinued due to a lack of efficacy**
			**MMP9 Andecaliximab** (GS-5745)	**2**	**Andecaliximab** (GS-5745)<br>Phase 1 gastroesophageal junction adenocarcinoma (2022) (Yoshikawa et al. 2022), Phase1 RA (Gossage et al. 2018), Phase3 gastric adenocarcinoma (https://clinicaltrials.gov/ct2/show/NCT02545504?term=Andecaliximab&phase=2&draw=2&rank=1), phase2/3 ulcerative colitis (https://clinicaltrials.gov/ct2/show/NCT02520284?term=Andecaliximab&phase=2&draw=2&rank=2)
			**Salvianolic acid B*** (SA-B)	**3**	**Salvianolic acid B*** (SA-B)<br>Preclinical fatty liver disease(https://clinicaltrials.gov/ct2/show/NCT05076058?term=Salvianolic+acid+B&draw=2&rank=2),<br>Hepatitis B (Liu et al. 2002), in

					vitro cells, and in vivo mice (Lin et al. 2007)
			**Curcumin***		**Curcumin***<br>Phase 1/2<br>RA, type 2 diabetes<br>(https://clinicaltrials.gov/ct2/show/NCT01052597),<br>(https://clinicaltrials.gov/ct2/show/NCT00752154?term=curcumin&cond=Rheumatoid+Arthritis&draw=2&rank=1)*,<br>(Dai et al. 2018),(Chuengsamarn et al. 2014)
			**MMP3: Prinomastat**		**Prinomastat**<br>Phase 3 lung and prostate cancer, brain and central nervous system tumours (https://clinicaltrials.gov/ct2/results?cond=&term=Prinomastat&cntry=&state=&city=&dist=)
			**Batimastat**		**Batimastat**<br>(Kumar et al. 2010) Duchenne muscular dystrophy (DMD), Internal resorption (IR),<br>Tuberculosis (TB) (Tang et al. 2022; Yu et al. 2022; Zhou et al. 2021)
			**Ilomastat**		**Ilomastat**<br>Leukaemia, Colon cancer, cancer-associated oedema (Wang et al. 2022; Zuo et al. 2022; Bissinger et al. 2021); Ohta et al. 2022)
			**ADAMTS4: US9206139, 5**		**US9206139, 5**<br>Rat study<br>Osteoarthritis<br>(Zhao et al. 2022)

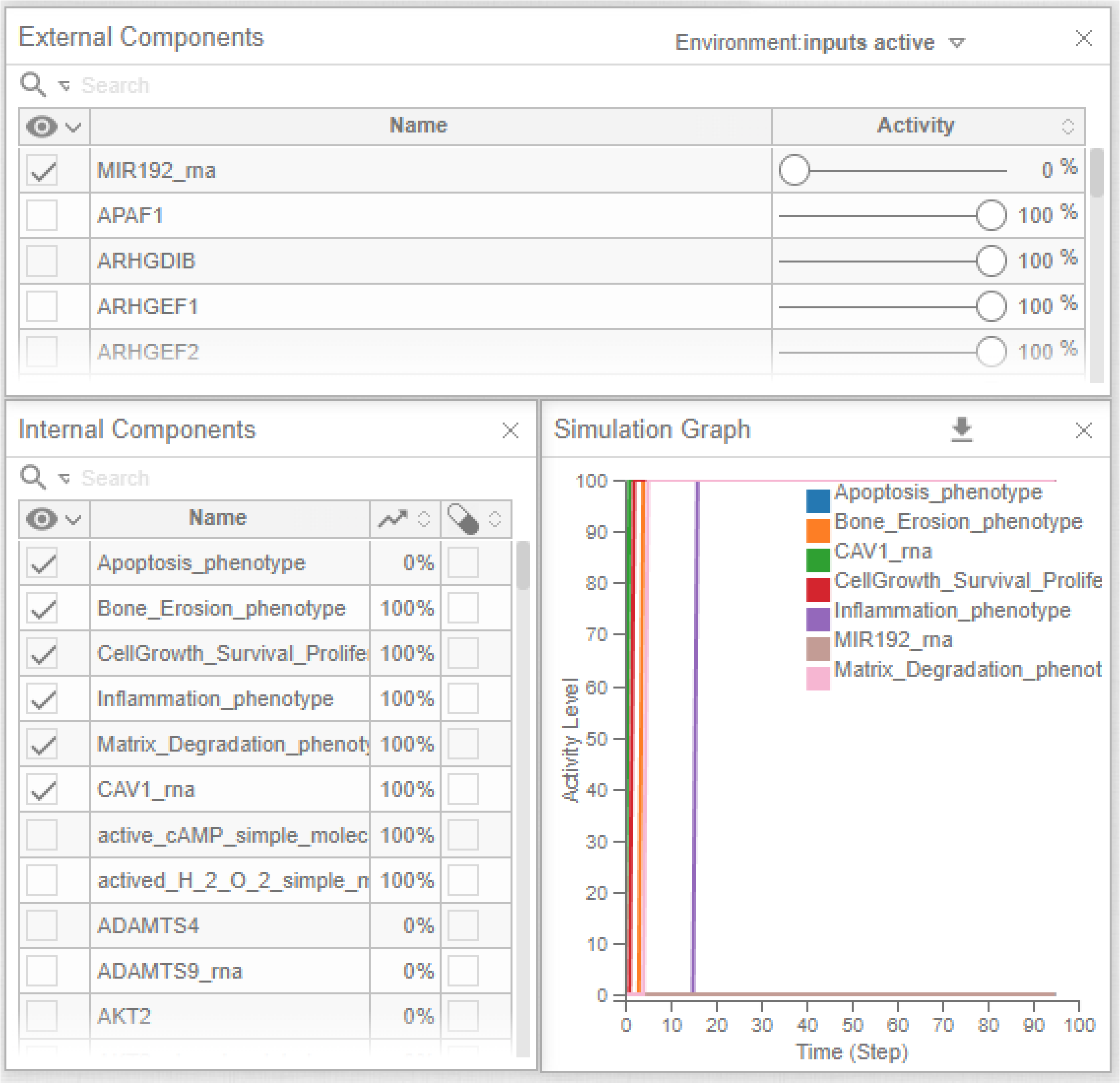

Supplementary Figure 3: Simulation results using the Cell Collective platform with the modified RA-FLS model. All inputs are kept active, and MIR192 is kept inactive. MIR192 is the inhibitor of CAV1, so when MIR192 is OFF, CAV1, a direct inhibitor of apoptosis, is activated. Under these conditions, apoptosis is OFF while all other phenotypes are ON, similar to the RA pathogenic state.

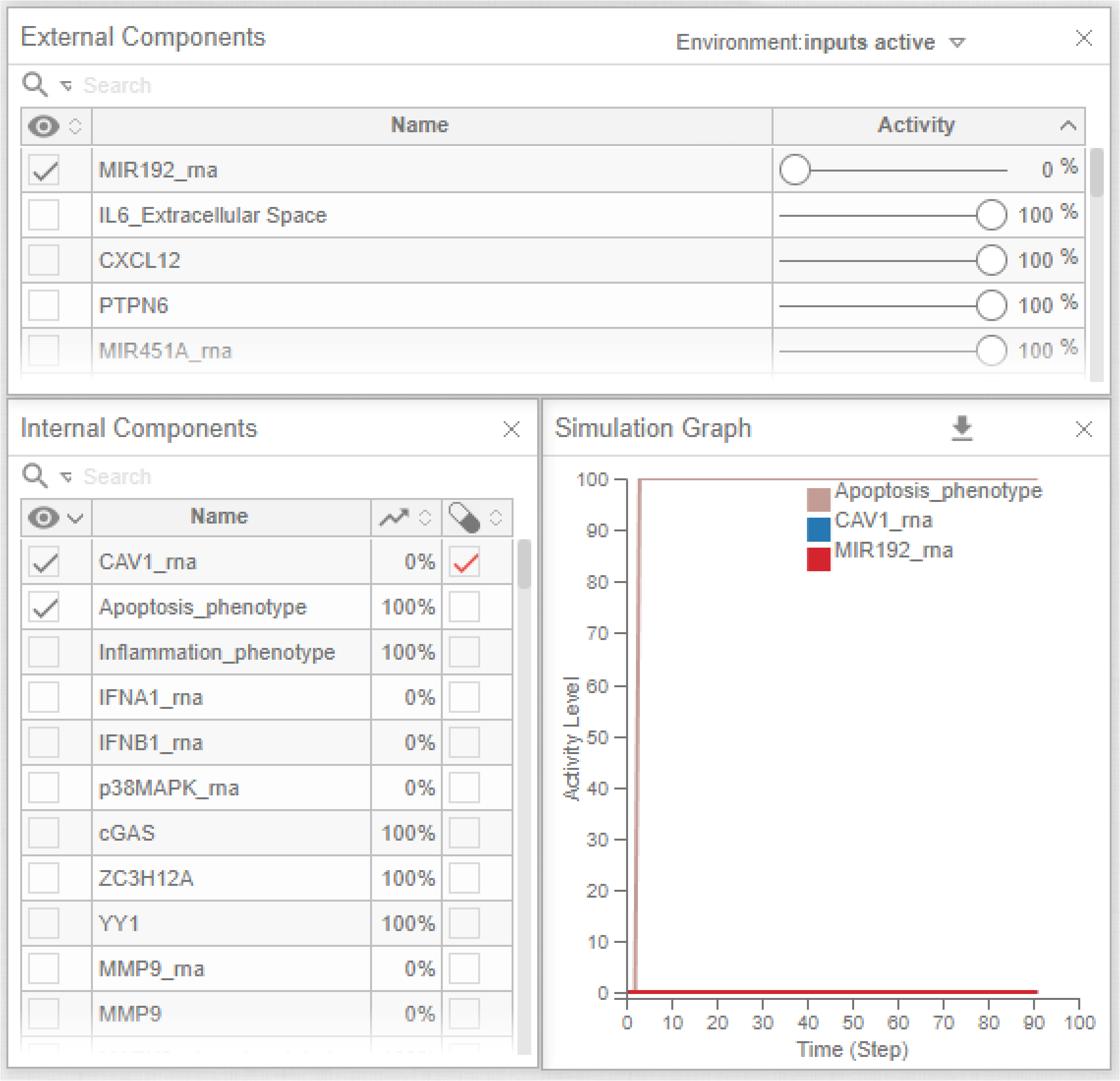

Supplementary Figure 4: Simulation results using the Cell Collective platform with the modified RA-FLS model. All inputs are kept active, and MIR192 is kept inactive. CAV1 is inhibited by Zoledronic acid (ZA) which results in the activation of apoptosis.

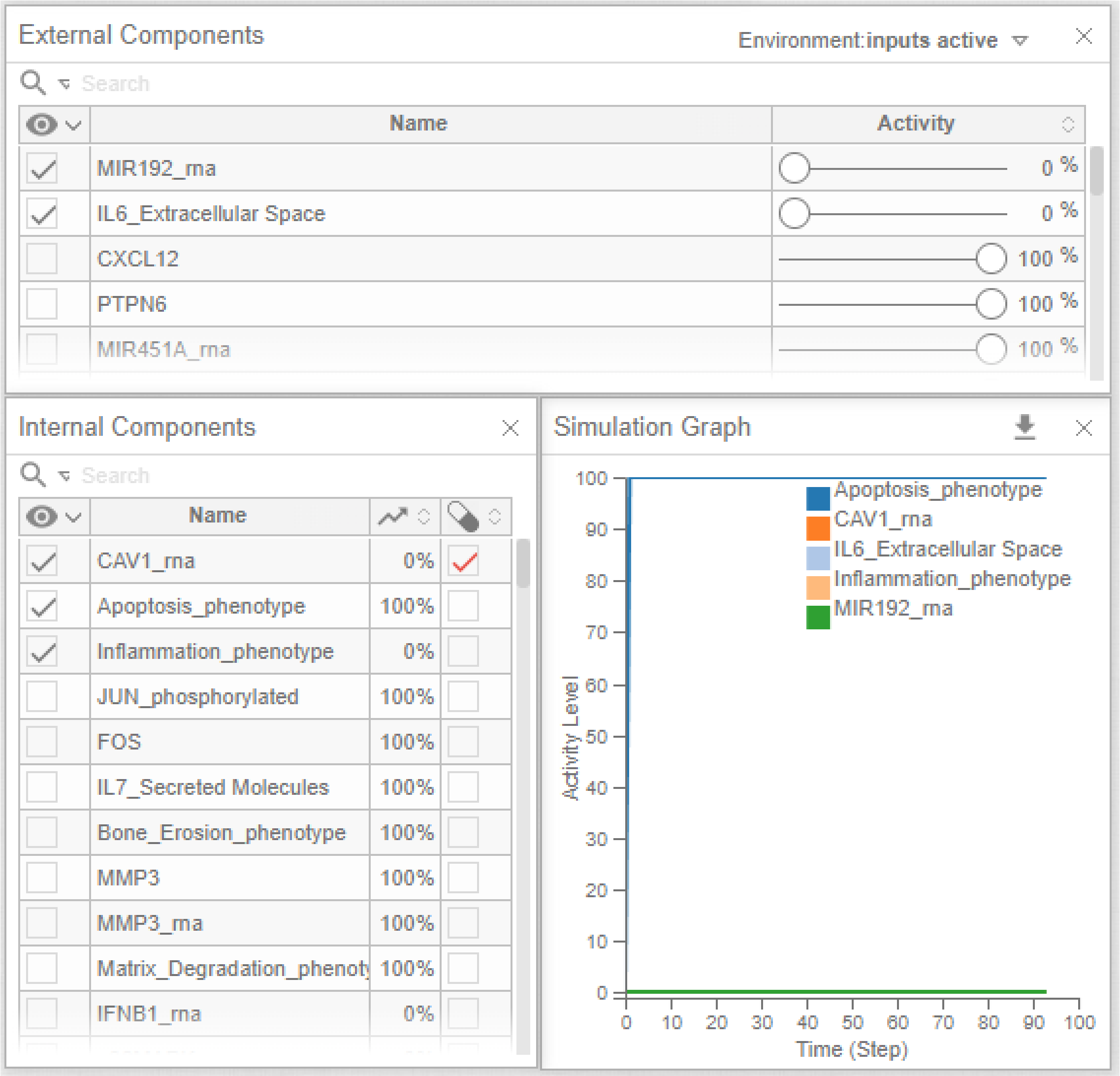

Supplementary Figure 5: Simulation results using the Cell Collective platform with the modified RA-FLS model. All inputs are kept active, and MIR192 is kept inactive. CAV1 is targeted by Zoledronic acid (ZA), and IL6 is targeted by Sarilumab, which results in the activation of apoptosis and the inhibition of inflammation.

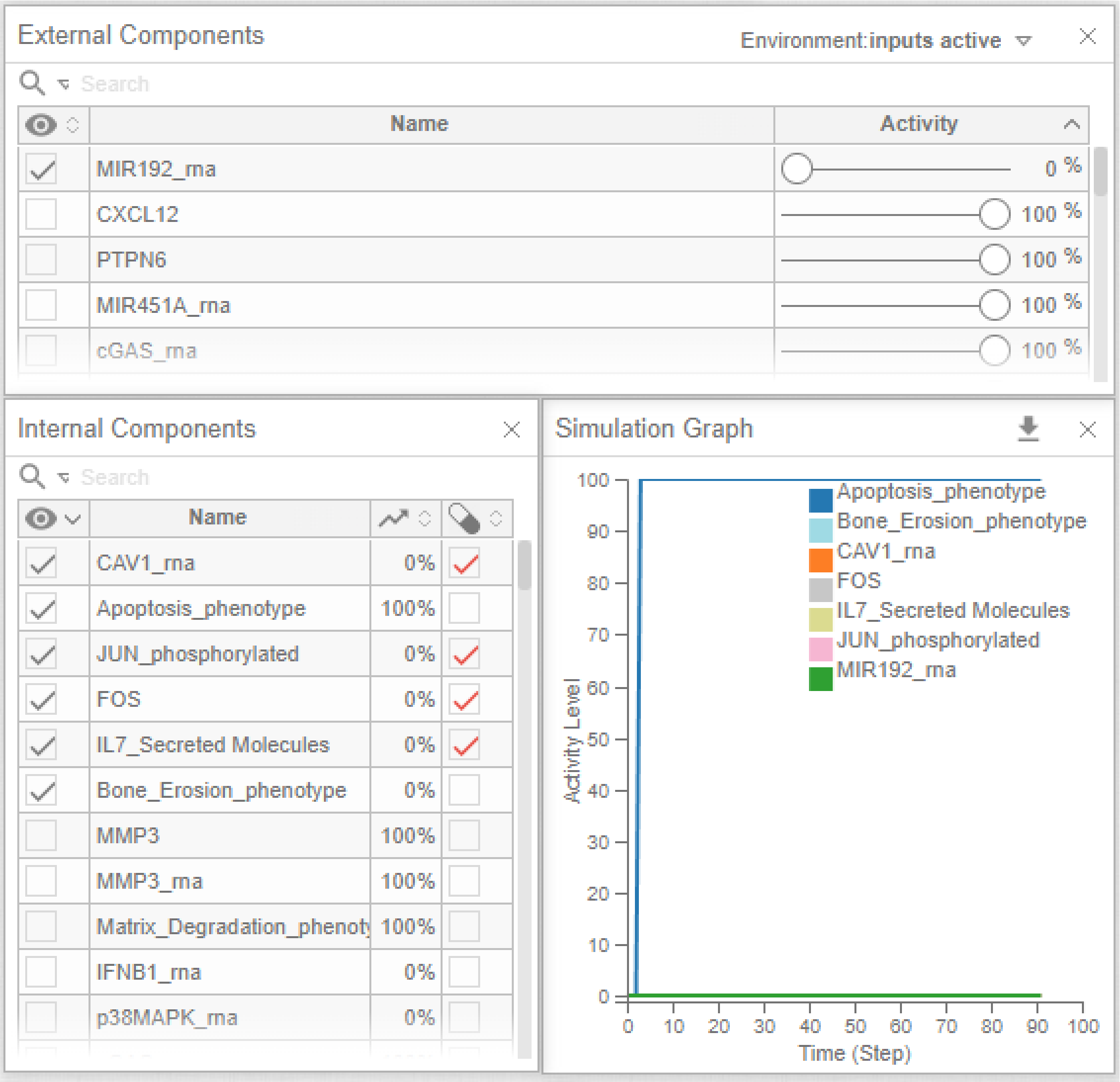

Supplementary Figure 6: Simulation results using the Cell Collective platform with the modified RA-FLS model. All inputs are kept active, and MIR192 is kept inactive. In addition, CAV1 is targeted by ZA, AP1 (Jun & Fos) is targeted by T-5224, and IL7 by GSK2618960 which results in the activation of apoptosis and the inhibition of bone erosion.

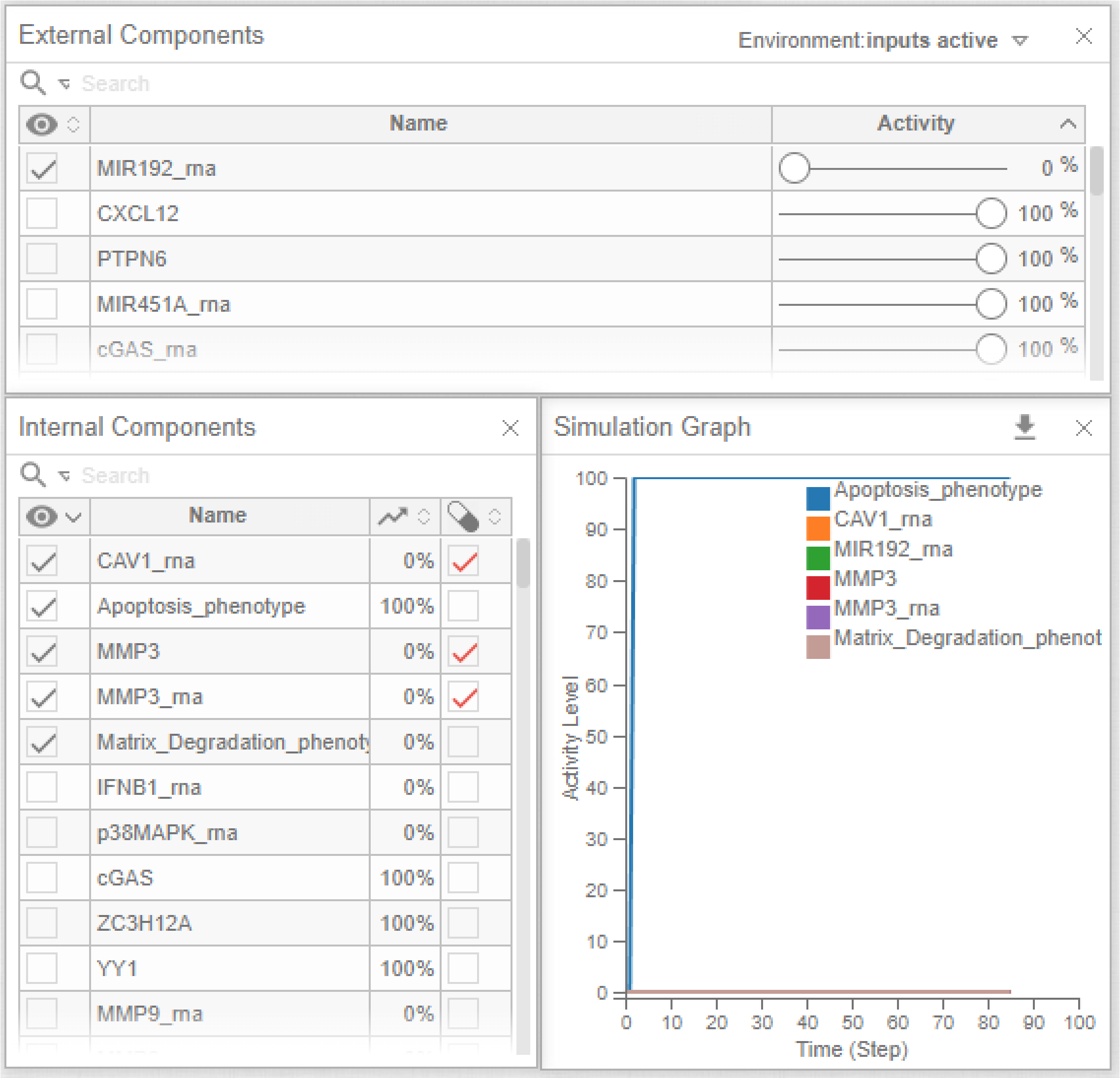

Supplementary Figure 7: Simulation results using the Cell Collective platform with the modified RA-FLS model. All inputs are kept active, and MIR192 is kept inactive. CAV1 is targeted by ZA, and MMP3 is targeted by Batimastat, which results in the activation of apoptosis and the inhibition of matrix degradation.

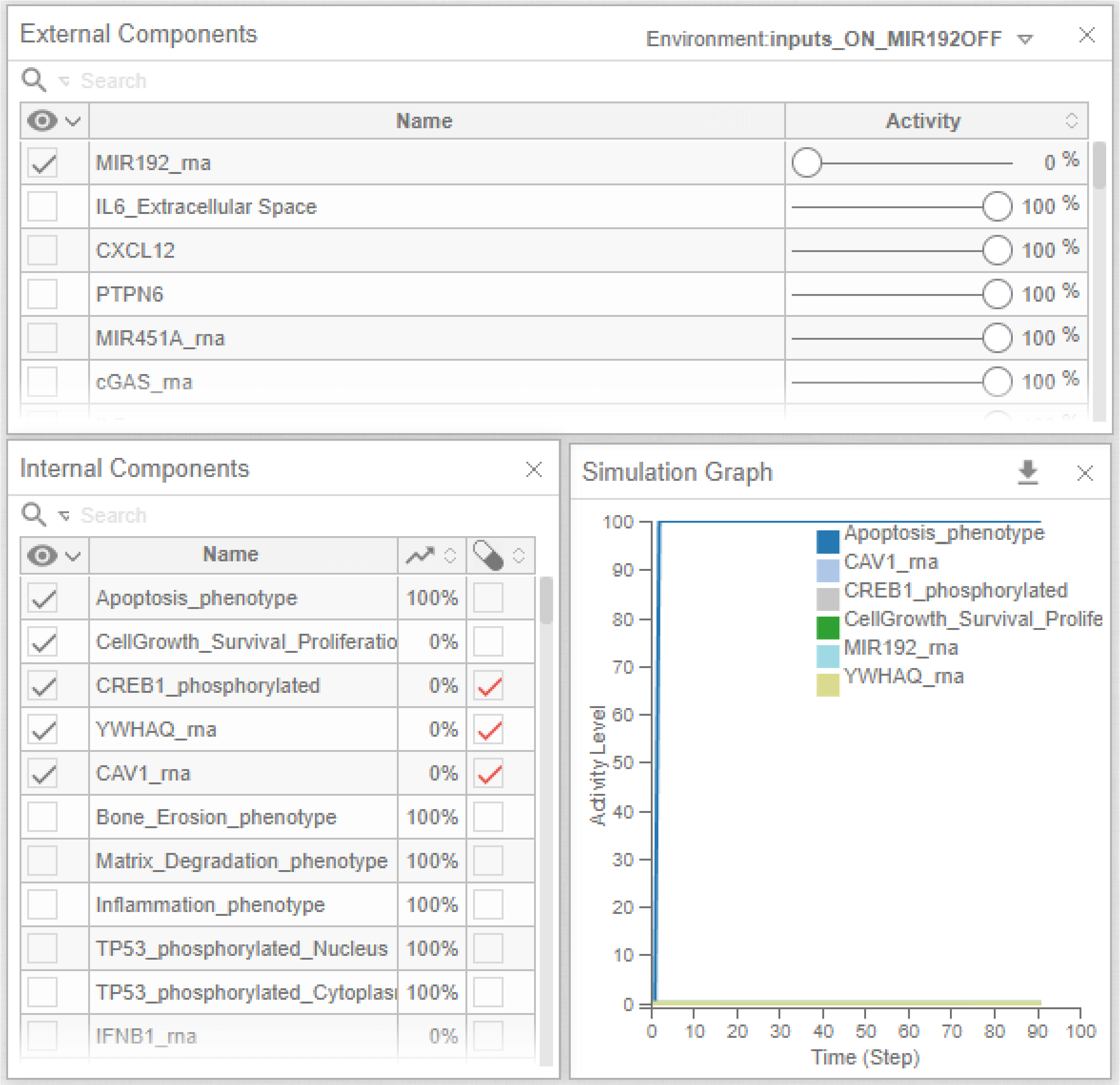

Supplementary Figure 8: Simulation results using the Cell Collective platform with the modified RA-FLS model. All inputs are kept active, and MIR192 is kept inactive. CAV1 is targeted by ZA, CREB1 is targeted by 666-15, and YWHAQ (via FOXO1) is targeted by AS1842856, which results in the activation of apoptosis and the inhibition of cell proliferation.

Supplementary Table 3: Biological_scenarios_modified_model_default1_CAV1_ON († displays RA-FLS specific scenarios reproduced with additional conditions of (* known biological behaviour, ** model conditions added to reproduce the RA-FLS biologi

Name	Inputs	Perturbations	Apoptosis_ph	CellGrowt	Bone_Eros	Matrix_De	Inflammation_phenotype
DEFAULT (Inputs 1)		CAV1_rna:1					
TNF KO	TNF_Extracellular_Space:0	CAV1_rna:1					0
IL6 KO	IL6_Extracellular_Space:0	CAV1_rna:1					0
IL17 KO	IL17_Extracellular_Space:0	CAV1_rna:1					0
FASFASLG ON		FASFASL_complex:1, CAV1_rna:1	1		1		
TNF ON_NFKB OFF	TNF_Extracellular_Space:1	NFKB_complex:0, CAV1_rna:1	1				
AKT2 ON_FASLG ON_BID OFF	FASLG:1	AKT2:1, BID:0, CAV1_rna:1	0				
TGFB1 ON_PIK3R5 ON		TGFB1:1, PIK3R5_phosphorylated:1, CAV1_rna:1	0	1			
PDGFA ON	PDGFA:1	CAV1_rna:1		1			
PDGFA ON_AKT2 ON	PDGFA:1	AKT2:1, CAV1_rna:1		1			
FOXO1 OFF		FOXO1:0, CAV1_rna:1		1			
WNT5A KO	WNT5A:0	CAV1_rna:1			0		
†WNT5A KO_IF_**SFRP5 ON	WNT5A:0, SFRP5:1	CAV1_rna:1			0		
SFRP5 ON	SFRP5:1	CAV1_rna:1			0		0
TNFSF11 ON	TNFSF11:1	CAV1_rna:1			1		
TNFSF11 KO	TNFSF11:0	CAV1_rna:1			0		
†TNFSF11 KO _IF_**SFRP5 ON	TNFSF11:0, SFRP5:1	CAV1_rna:1			0		
TNF ON	TNF_Extracellular_Space:1	CAV1_rna:1					1
†TNF ON _IF_*NFKB ON	TNF_Extracellular_Space:1, IKBANF	CAV1_rna:1					1
MMP1 OFF		MMP1:0, CAV1_rna:1				0	
MMP9 ON		MMP9:1, CAV1_rna:1				1	

Supplementary Table 4: Biological_scenarios_modified_model_default0 († displays RA-FLS specific scenarios reproduced with additional conditions of (* known biological behaviour, ** model conditions added to reproduce the RA-FLS biological scenarios. Th

Name	Inputs	Perturbations	Apoptosis_	CellGrowtl	Bone_Eros	Matrix_De	Inflammation_phenotype
DEFAULT (Inputs 0)							
TNF KO	TNF_Extracellular_Space:0						0
IL6 KO	IL6_Extracellular_Space:0						0
IL17 KO	IL17_Extracellular_Space:0						0
FASFASLG ON		FASFASL_complex:1	1		1		
†FASFASLG ON_IF_**MIR192 ON (modified)	MIR192_rna:1	FASFASL_complex:1	1		1		
TNF ON_NFKB OFF	TNF_Extracellular_Space:1	NFKB_complex:0	1				
† TNF ON_NFKB OFF_IF_**MIR192 ON (modified)	TNF_Extracellular_Space:1, MIR192_rna:1	NFKB_complex:0	1				
AKT2 ON_FASLG ON_BID OFF	FASLG:1	AKT2:1, BID:0	0				
TGFB1 ON_PIK3R5 ON		TGFB1:1, PIK3R5_phosphorylated:1	0	1			
PDGFA ON	PDGFA:1			1			
PDGFA ON_AKT2 ON	PDGFA:1	AKT2:1		1			
FOXO1 OFF		FOXO1:0		1			
WNT5A KO	WNT5A:0				0		
†WNT5A KO_IF_**SFRP5 ON	WNT5A:0, SFRP5:1				0		
SFRP5 ON	SFRP5:1				0		0
TNFSF11 ON	TNFSF11:1				1		
TNFSF11 KO	TNFSF11:0				0		
† TNFSF11 KO_IF_**SFRP5 ON	TNFSF11:0, SFRP5:1				0		
TNF ON	TNF_Extracellular_Space:1						1
†TNF ON_IF_*NFKB ON	TNF_Extracellular_Space:1, IKBANFKB1RELA_complex:1						1
MMP1 OFF		MMP1:0				0	
MMP9 ON		MMP9:1				1	
